# Exposure limits for indoor volatile substances concerning the general population: The role of population-based differences in sensory irritation of the eyes and airways for assessment factors

**DOI:** 10.1007/s00204-023-03642-w

**Published:** 2024-01-19

**Authors:** Stefan Kleinbeck, Peder Wolkoff

**Affiliations:** 1 Leibniz Research Centre for Working Environment and Human Factors, Dortmund, Germany; 2 National Research Centre for the Working Environment, Copenhagen, Denmark

**Keywords:** Age, Assessment factor, Children, Gender, Sensitivity, Sensory irritation

## Abstract

Assessment factors (AFs) are essential in the derivation of occupational exposure limits (OELs) and indoor air quality guidelines. The factors shall accommodate differences in sensitivity between subgroups, i.e., workers, healthy and sick people, and occupational exposure versus life-long exposure for the general population. Derivation of AFs itself is based on empirical knowledge from human and animal exposure studies with immanent uncertainty in the empirical evidence due to knowledge gaps and experimental reliability. Sensory irritation in the eyes and airways constitute about 30–40% of OELs and is an abundant symptom in non-industrial buildings characterizing the indoor air quality and general health. Intraspecies differences between subgroups of the general population should be quantified for the proposal of more ‘empirical’ based AFs. In this review, we focus on sensitivity differences in sensory irritation about gender, age, health status, and vulnerability in people, based solely on human exposure studies. Females are more sensitive to sensory irritation than males for few volatile substances. Older people appear less sensitive than younger ones. However, impaired defense mechanisms may increase vulnerability in the long term. Empirical evidence of sensory irritation in children is rare and limited to children down to the age of six years. Studies of the nervous system in children compared to adults suggest a higher sensitivity in children; however, some defense mechanisms are more efficient in children than in adults. Usually, exposure studies are performed with healthy subjects. Exposure studies with sick people are not representative due to the deselection of subjects with moderate or severe eye or airway diseases, which likely underestimates the sensitivity of the group of people with diseases. Psychological characterization like personality factors shows that concentrations of volatile substances far below their sensory irritation thresholds may influence the sensitivity, in part biased by odor perception. Thus, the protection of people with extreme personality traits is not feasible by an AF and other mitigation strategies are required. The available empirical evidence comprising age, lifestyle, and health supports an AF of not greater than up to 2 for sensory irritation. Further, general AFs are discouraged for derivation, rather substance-specific derivation of AFs is recommended based on the risk assessment of empirical data, deposition in the airways depending on the substance’s water solubility and compensating for knowledge and experimental gaps. Modeling of sensory irritation would be a better ‘empirical’ starting point for derivation of AFs for children, older, and sick people, as human exposure studies are not possible (due to ethical reasons) or not generalizable (due to self-selection). Dedicated AFs may be derived for environments where dry air, high room temperature, and visually demanding tasks aggravate the eyes or airways than for places in which the workload is balanced, while indoor playgrounds might need other AFs due to physical workload and affected groups of the general population.

## Introduction

Exposure limits for volatile substances should be based on risk assessments considering empirical research (World Health Organization 2010). Such knowledge is a snapshot of current and past research that might be challenged by future and conflicting research results (e.g., based on newly developed research methods). Therefore, exposure limits should be regarded as temporary, since new empirical and reliable research data will require a reassessment.

Risk assessment should consider—on the one hand—the terms and conditions of exposure and—on the other hand—all exposed groups of people. In the worst case, exposure to a volatile substance lasts 24 h a day, seven days a week for a span of life (Rohlman et al. 2008; World Health Organization 2010). Further, one must consider that certain subgroups in the general population (might) differ systematically in sensitivity. Individual differences in sensitivity might be due to internal as well as external factors. Accordant to others (Bell et al. 2013; Hooper and Kaufman 2018; Portier et al. 2010), susceptibility of an individual, here, refers to factors inherent to internal factors (physical predisposition, i.e., internal defects) and vulnerability refers to external factors (e.g., low humidity leads to dry eyes in an otherwise healthy person). Both susceptible and vulnerable conditions could increase the sensitivity to volatile substances. The distinction, however, is far from clear as every perception is the result/interaction of internal and external factors. Susceptibility and vulnerability are often used interchangeable (cf., Merriam-Webster Thesaurus of ‘vulnerability’^1^). Getting back to the general population, i.e., children, older people, or sick people might be more vulnerable or susceptible to certain volatile substances than healthy adults (Rohlman et al. 2008; World Health Organization 2010) and could, therefore, be more sensitive as a group. Such ‘group sensitivities’ must be considered in risk assessment.

Experimental exposure studies with human subjects are considered the gold standard for the derivation of No Observable Adverse Effect Concentrations for sensory irritation (Brüning et al. 2014; Nielsen and Wolkoff 2017). However, as experimental human exposure studies with susceptible or vulnerable people (i.e., children and persons with moderate or severe diseases) are rare from an ethical standpoint, other knowledge must be considered for risk assessment.

Much knowledge about exposure limits was derived from experimental animal models. In the past, animal models have provided information about the ‘mode of action’ (MOA) of different volatile substances (Andersen and Dennison 2001; Bushnell et al. 2007; Clewell 2005). However, the use of data from animal studies for setting human exposure limits is complicated by interspecies differences. Meanwhile, the current strategy in toxicology is to reduce and replace animal testing (National Research Council 2007). Therefore, alternative methods are propagated that use less animals, such as Quantitative Structure Activation Relations (QSAR; OECD 2014; Sullivan et al. 2014), and this also helps to establish a MOA/Adverse Outcome Pathways (AOP) (Organisation for Economic Co-operation and Development; OECD 2014; Patlewicz et al. 2014).

Since about 30–40% of the occupational exposure limits (OELs) (Dick and Ahlers 1998; Paustenbach 2001) of volatile substances are set to avoid irritation of mucous membranes, sensory irritation is an appropriate and sensitive parameter for measuring irritating effects in the respiratory tract (Brüning et al. 2014). Chemical sensitivity spanning chemosensory modalities seems unlikely (Lundström et al. 2012), thus different target sites (Alarie 1973; Arts et al. 2002) must be investigated. Shusterman (2002) described levels of impact on mucous membrane irritation depending on the water solubility of the volatile substance in the airways, and considering that the sites of irritation are concentration dependent, i.e., a nasal irritant at low concentration could readily become a pulmonary irritant at higher concentration (cf., U. S. Environmental Protection Agency 1994):

- Eyes, nose, pharynx, larynx (high water solubility) – high deposition.
- Trachea, bronchi (medium water solubility) – medium deposition.
- Bronchioles, alveoli (low water solubility) – low deposition.

The first sites of contact with a volatile substance in the air are the eyes and the nasal mucosa, which are the focus of most of the methods assessing sensory irritation (Beuerman and Stern 2005; Doty et al. 2004; Kjærgaard et al. 1992). The cornea of the eye is the most innervated tissue in the body (Bonini et al. 2003; Yang et al. 2018; Zander and Weddell 1951). Various stimuli can be transduced by the cornea: thermal, mechanical, and chemical (Beuerman and Stern 2005; Yang et al. 2018). About 70% of the receptors are polymodal nociceptors, which convey inter alia pain in response to chemical stimuli via unmyelinated C-type nerves (Steen and Reeh 1993; Yang et al. 2018). Furthermore, 10% of the receptors in the cornea are Aδ- and C-fiber cold receptors, which may react (amongst others) to environmentally (e.g., dry air) induced tear film desiccation (Acosta et al. 2001; Beuerman and Stern 2005; Yang et al. 2018). Sensory irritation of the upper and lower airways is mediated by the activation of unmyelinated axons of the trigeminal nerve in the nasal mucosa (Alarie 1973) and beneath the epithelium of the lower airways (Benemei et al. 2015; Geppetti et al. 2010; Narula et al. 2014). Chemoreceptors/nociceptors (e.g., transient receptor potential channels; TRP channels) located on free endings of peripheral nerves innervating the airways (Bessac and Jordt 2008) elicit various reflexes or defense mechanisms. Therefore, there are receptors in the eyes, the nose, and lower airways that can be activated by volatile substances (Lehmann et al. 2017), which results in specific perceptions (e.g., burning, stinging; cf., Hummel 2000) and other reflexes and defense mechanisms, e.g., eye blinking (Doty et al. 2004). The response cascade elicited by volatile substances is of special interest because it allows for identifying objective markers of sensory irritation. A scientific approach to describe a response cascade is the concept of AOP (Ankley et al. 2010). Thus, Martinez and Eling (2019) describe such an AOP with a molecular-initiating event (MIE) and three sequential key events (KE) leading to an adverse outcome (sensory irritation).

A prolonged exposure to volatile substances eliciting sensory irritation (so-called local irritants; Schaper 1993) can lead to inflammation and subsequent tissue damage (cytotoxic effect) (Brüning et al. 2014). Thus, physiological indicators of sensory irritation should be avoided for the protection of the organism from chronic health effects such as tissue damage; these indicators are trigeminal-mediated reflexes (e.g., increase of eye blinking frequency) or inflammation (e.g., release of neuropeptides such as substance P) (Brüning et al. 2014). The avoidance of sensory irritation is one aim of setting exposure limits (Brüning et al. 2014; Nielsen and Wolkoff 2017). Thus, the difficulty of distinguishing between genuine sensory irritation-mediated perceptive and objective effects and cytotoxic effects should be emphasized.

The purpose of risk assessment is to identify a level of exposure to volatile substances at which the freedom of health effects is provided with some assurance (health-based standard; Fairhurst 1995). There are several critical outcomes of volatile substances that must be considered in setting exposure limits (World Health Organization 2010). If the database is limited, several (n-fold) assessment factors are used for extrapolation; for example, from animal exposure studies to humans or from young healthy individuals to the general population (Fairhurst 1995). The nomenclature of such factors differs depending on the organization (cf., Dankovic et al. 2015): Assessment factors, extrapolation factors, safety factors, uncertainty factors. This review uses the term assessment factors (AF, according to Vermeire et al. 1999, p. 441) and aims at intra-species extrapolation from young, healthy, mostly male individuals to the general population. Such an AF is’inherently arbitrary, debatable and potentially variable, depending on particular circumstances’ (Fairhurst 1995, p. 379).

While OELs usually are set to protect the worker population at employable age, environmental and indoor exposure limits should protect the general (whole) population including susceptible and vulnerable sub-groups. For the worker population, individual variability in sensitivity to sensory irritation is considered by a maximum AF of 2 if the threshold is based on valid human exposure studies with enough subjects (Brüning et al. 2014; Fairhurst 1995; Nielsen and Wolkoff 2017). Such an AF could be higher for the general population, comprising young and old, females, males, children, individuals with allergy (asthma) and other diseases (which may alter the sensitivity to sensory irritants). One reason is that the relatively low number of subjects in the exposure studies is not representative of the whole spectrum of human variability. Thus, for the general population usually an AF of 10 shall be applied according to ECHA Guidance R8 (Annex R.8–15: Guidance on Derivation of DNEL/DMEL from Human Data).

The assessment/uncertainty factor of 10 for the general population, however, is not dedicated to specific studies (endpoints) that aim to derive such AFs. Thus, the first aim of this study is to identify relevant controlled human exposure studies that could be a platform for derivation of (an) AF(s) for the general population, which is specific for ‘sensory irritation’ in the eyes and airways. The second aim is to propose (an) adequate AF(s) for the general population based on the identified exposure studies. Such a factor still has the disadvantage of AFs per se (cf., Fairhurst 1995), but its derivation is a step towards a more evidence-based approach to risk assessment (for sensory irritation).

## Procedure

A literature search was carried out to identify relevant human experimental exposure studies. A total of more than 400 papers on sensory irritation in humans was scanned for information about (human) intraspecies variance in sensitivity to sensory irritation in the eyes and airways. Sensory irritation, here, comprises stimulation of nociceptors located in the upper airways. Such afferent stimulation may cause 'pungent sensations' to limit cytotoxic exposure but may also stimulate protective physiological responses suited to mitigate tissue injury (see discussion by Nielsen and Wolkoff 2017 and OECD 2017).

### Grouping factors of the general population

In search of potential sources of intraspecies variability in sensory irritation, the following grouping factors for the general population were considered:

1. Gender
2. Age

- Children.
- Older people/retired.

(3) Health status

- People with allergic diseases.
- Asthmatics.

(4) Vulnerability.^2^

- (self-reported) multiple chemical sensitivity (MCS).
- Psychological traits (e.g., affectivity).

Several studies have been identified with respect to the variability of sensory irritation in these sub-groups. The quality of studies was evaluated regarding the criteria for controlled chamber studies described by Nielsen and Wolkoff (2017). The most important criterion is that sensory irritation was appropriately measured, and the volatile substance exposure was well characterized.

Sensory irritation is linked to pungent sensations: stinging, piquancy, burning, tingling, freshness, prickling, irritation, itching, and cooling (Doty et al. 2004). Pungency refers to nasal (and oral) sensations that are mediated by the trigeminal nerve (Doty et al. 2004). As stated above, there are different target sites of sensory irritation in humans (Doty et al. 2004; Shusterman 2002). The following examples (non-exclusive) of markers of irritation are used at these target sites:

- Eyes: i.e., irritation thresholds, blinking frequency, break-up time, eye redness, biomarkers in tear film, rating of eye irritation.
- Nose: i.e., irritation thresholds, nasal airflow, changes in secretion, biomarkers in nasal lavage fluid, changes in nasal blood flow, changes in ciliary beat frequency, mucociliary clearance rate, rating of nasal irritation.
- Pharynx/Larynx; Throat: i.e., changes in blood flow, rating of throat irritation, exhaled nitric oxide (FeNO).
- Trachea/Bronchi: i.e., coughing, breathing parameters, exhaled nitric oxide (FeNO), urge to cough.
- Bronchioles/Alveoli: i.e., coughing, breathing parameters, exhaled nitric oxide (FeNO), urge to cough, exhaled breath condensate.
- The dry sensation in eyes and upper airways is often associated with exposure to some sensory irritants, which may be considered a proto-state of sensory irritation as proposed by Cain et al (2008). However, dry eye sensation may also be induced by other routes, e.g., desiccation of the eye tear film (Wolkoff 2020) or airways (Wolkoff 2018).

### Subjective vs. objective markers of sensory irritation

Markers of sensory irritation are not necessarily assigned to only one level of sensory irritation (like breathing parameters). For this study, the level of (subjectively) perceived irritation is of secondary importance. Usually, objective markers are more reliable than ratings (Arts et al. 2006a; Paustenbach et al. 1997; Philpott et al. 2006) as the latter combine sensory and cognitive odor/irritation processing (Cometto-Muñiz and Cain 1991; van Thriel et al. 2008); thus, **objective markers** are **prioritized**. For example, the lateralization of chemicals (basis for irritation threshold studies) can only occur if an irritation takes place (e.g., Dalton et al. 2000) and this irritation is due to the volatile substance (chemesthesis). However, other stimuli than sensory irritants may influence sensory irritation. For instance, reflexive changes in eye blinking frequency can also be influenced by mechanical impact, thermal changes, air dryness, visual tasking, and individual factors (Klenø and Wolkoff 2004; Wolkoff 2020, 2018, 2017; Yang et al. 2018). Objective markers may be influenced by other than chemical exposure. Examples are biomarkers in nasal lavage and congestion (i.e., inflammation, allergy; Guilemany et al. 2009), coughing (i.e., air temperature; Benemei et al. 2015), and breathing frequency/depth (i.e., air temperature, COPD; Barry and Annesi-Maesano 2017). Therefore, potential stimuli having an impact on markers of irritation must be controlled in the experimental studies to trace back the sensory irritation to the chemical exposure without invalidating bias. Accordingly, information about different markers of sensory irritation is important.

For subjective measures, the time course of rated sensory irritation provides indirect information of sensory irritation, which takes place, though odor perception may increase the report of symptoms (Nielsen et al. 2007; Wolkoff et al. 2006) as shown by Cometto-Muñiz and Cain (1991). This is because odor thresholds generally are one to three orders of magnitude lower than thresholds for sensory irritation (Cometto-Muñiz and Abraham 2016). Contrary to odor ratings that generally show adaptation over time, ratings of sensory irritation should reveal an over-time increase in perceived irritation to a certain plateau (van Thriel et al. 2002). For eye irritation, many studies indicate that the time course of ratings (over hours) is close to objective markers (i.e., eye blinking frequencies; Juran et al. 2012; Kleinbeck et al. 2017, 2008; Pacharra et al. 2016c). Furthermore, the time course of eye irritation ratings appears plausible in view of pharmacokinetic/pharmacodynamics (PK/PD) modeling^3^ of the rabbit eye (Kleinbeck et al. 2020). Thus, **subjective ratings** will also be **considered with adequate caution**, especially in studies where sensory irritation is also measured by objective markers.

There is only one experimental exposure study with children (Hummel et al. 2007); thus, their possible susceptibility or vulnerability for sensory irritation in the eyes and respiratory tract must be assessed by other approaches. Childhood can be conceptualized as a sequence of life stages comprising of windows of susceptibility to environmental agents, which might be enhanced (Firestone et al. 2008; Saadeh and Klaunig 2015; U. S. Environmental Protection Agency 2005). For this reason, developmental aspects of the target organs and their respective innervation will be considered. Potential factors for differential sensitivity in children compared to adults are the eye (precorneal) tear film stability (Borchman et al. 2012; Shrestha et al. 2011; Sledge et al. 2017), intranasal trigeminal function (Hummel et al. 2007), inhalation dosimetry (Foos et al. 2008; Garcia et al. 2009; Ginsberg et al. 2005), specific health effects and risks (Nielsen et al. 2013; Selgrade et al. 2008; Sunderland et al. 2019; Wolkoff and Nielsen 2010), internal dose metrics (Firestone et al. 2008; Valcke and Krishnan 2011), somatosensory processing (Uppal et al. 2016), and AOPs for developmental neurotoxicity (cf., Pistollato et al. 2020).

### Manifestations of sensitivity

Sensitivity in sensory irritation to volatile substances has different manifestations. First, sensitivity can refer to sensory irritation at different concentrations of a volatile substance (that is, a lower threshold of sensory irritation means higher sensitivity). Second, the same concentration of a volatile substance can elicit stronger reactions in the sensory system in sensitive than in ‘normal-healthy’ persons (that is, the same concentration leads to a stronger physiological reaction of the sensory system in sensitive people). Third, the same physiological reaction can be perceived differently due to cognitive processing, e.g., odor-mediated. It is unclear whether these manifestations are interrelated (at least, the third seems to be another entity), but it is possible that they are independent. At first glance, the first manifestation of sensitivity might be the most important in deriving exposure limits based on sensory irritation. At long-lasting exposures to volatile substances, however, the other manifestations may become relevant, too. For example, sensitization could occur at lower concentrations in more sensitive people (second manifestation), independently of their sensory irritation threshold. The third manifestation of sensitivity is the most difficult for the derivation of AFs. This manifestation of sensitivity can lead to (reports of) perceptual aspects of sensory irritation in the presence of a volatile substance but without detectable physiological markers of sensory irritation. Though this manifestation of sensitivity is primarily based on cognitive processing it can elicit (psychogenic) symptoms that could be unbearable for an affected person (e.g., with multiple chemical sensitivity (MCS)). An AF for such manifestation of sensitivity is difficult to derive without detailed knowledge about the causality.

### Temporal aspects of sensory irritation

Methods to identify differences in sensory irritation in humans are linked to different exposure durations. The toxic load in sensory irritation is a result of both concentration (C) and time (t) of exposure (C^n^ x t; Pauluhn 2019). While an exposure of a few seconds is reasonable for the assessment of (acute) thresholds, short-term exposure studies usually last minutes to hours, and epidemiological studies refer to years. However, temporal summation and carry-over effects may occur for sensory irritation: across seconds (Wise et al. 2009a, b, 2006, 2005), across minutes (Cain and Cometto-Muñiz 1995), and across hours (Cain et al. 2010; Cain and Cometto-Muñiz 1995; Kleinbeck et al. 2020, 2017).

Though differences in sensitivity might be observed in an exposure over seconds, a temporal summation over minutes or hours might occur at lower concentrations. The trigeminal system seems to act as a mass detector rather than a concentration detector (Frasnelli et al. 2017; Hummel and Frasnelli 2019; Kleinbeck et al. 2020). The detection of the trigeminal impact of a volatile substance may take tens of minutes especially at low concentrations (Cain et al. 2010; Wolkoff and Nielsen 2010). This is due to the latency of response of the responsible chemoreceptors for sensory irritation that could exceed minutes (as in the case of formaldehyde; Tian et al. 2009). Here, the second manifestation of sensitivity (see ‘Manifestations of sensitivity’) may play a role. Studies across days with human subjects are rare. Thus, a carry-over effect from one day to the other could not be found for ethyl acrylate in humans (Kleinbeck et al. 2020); however, signs of sensory irritation could be observed on every single day (increase in eye blinking frequency). Furthermore, Wolkoff et al. (2012) did not find an increase in sensory irritation in mice from day to day when repeatedly exposed to irritating mixtures of ozone-initiated limonene oxidation products over a period of ten days. These studies indicate that clean air between chemical exposures allowed for reversibility between daily impacts. However, the influence of longer continuous exposure periods (days) without recreation phases (which must be considered for indoor exposure limits) has not been studied in human subjects, yet.

Repeated exposure for three weeks to a water aerosol of 76 mg/m^3^ (31 ppm) acetic acid caused a substance-specific decrease in both the psychophysical and electrophysiological response to sensory irritation (n = 12) (Dalton et al. 2006). The study suggests that sensory irritation at low exposure levels either shows no carry-over effect or a carryover effect may cause desensitization; this is supported by a mice study, cf., Wolkoff et al. (2012), see above.

Epidemiologic studies comprising years were excluded from this review due to multi-factorial influences, and inadequate exposure characterization.

Highest to lowest priority for studies in this analysis of exposure:

1. across hours.
2. across minutes.
3. across seconds (thresholds).
4. across days.
5. across years (epidemiological studies) – excluded.

Therefore, studies will, in the first step, be tabulated by exposure duration (pattern) and target organ (cf., Shusterman 2002).

### Limitations with respect to volatile substances

Studies with carbon dioxide were excluded for the derivation of an AF due to experimental caveats (e.g., unrealistic high levels) and its acidic nature, though some of these studies show significant differences. However, the caveats hamper a reasonable transfer of results to risk assessment for sensory irritants. Excluded studies concern gender, age, and health status (Acosta et al. 2006; Cometto-Muñiz and Noriega 1985; Feng and Simpson 2003; Kjærgaard et al. 1992; Scheibe et al. 2009; Shusterman and Balmes 1997; Shusterman et al. 2003a).

## Influence of grouping factor *gender* on sensory irritation

Table 1 provides an overview of studies, which included ‘sensory irritation’ in both female and male subjects. Further characteristics of the studies (substances, substance delivery, examined concentrations, water solubility, and measures of sensory irritation) can be found in Sect. ‘Characteristics of the reviewed studies’ (Table 7). 4 At first glance, most studies either investigate exposure times of seconds (mostly threshold studies) or exposure times of hours. While the first kind of study utilizes acute irritation to determine irritation thresholds, the second kind of study usually uses lower concentrations. Sensory irritation, however, could arise by temporal summation in these studies. Therefore, it is essential to ensure that sensory irritation occurs at all during studies with hours of low exposure concentrations. Therefore, only studies with objective signs of irritation at the highest investigated concentration were analyzed.

**Table 1:** Studies of sensory irritation in males and females (bold number, if significant gender difference is observed)

	Exposure duration							
	Hours		Minutes		Seconds		Days	
	Objective	Subjective	Objective	Subjective	Objective	Subjective	Objective	Subjective
Eyes	[1] [2] [3] [4] [5] [6] [7] [8] [9] [10] [11] [12]	[1] [2] [3] [4] [5] [6] [7] [8] [9] [10] [11] [12]	[14] [15]	[15]			[29] [30]	[29] [30]
Nose	[1] [5] [6] [7] [8] [11] [12] [13]	[1] [2] [3] [4] [5] [6] [7] [8] [10] [11] **[12]** [13]			**[16]** [17] [18] [19] [20] [21] [**22**] [23] [24]	[16] [17] **[20]** [23] **[25]** [26] [27]	[29] [30]	[29] [30]
Pharynx/Larynx; Throat	**[1]**	**[1]** [2] [3] [4] [5] [6] [7] [8] [10] [11]						
Trachea/Bronchi		**[1]** [2] [3] [4] [5] [6] [7] [8] [10] [11]			**[28]**^**a**^			
Bronchioles/Alveoli								

^a^Stimulus is thermal pain

**Hours**

**[1]** Ernstgård et al. (2002) *n* = 56

[2] Gminski et al. (2011); *n* = 24

[3] Hey et al. (2009); *n* = 23

[4] Juran et al. (2012); *n* = 24

[5] Kleinbeck et al. (2008); *n* = 24

[6] Kleinbeck et al. (2017); *n* = 19

[7] Pacharra et al. (2017); *n* = 37

[8] Pacharra et al. (2016c); *n*= 48

[9] Schäper et al. (2015); *n*= 32

[10] Sucker et al. (2019); *n* = 22

[11] van Thriel et al. (2010); *n* = 16

**[12]** Wålinder et al. (2008); *n* = 29

[13] Sundblad et al. (2004); *n*= 12

**Minutes**

[14] Shusterman et al. (2005); *n* = 16

[15] Yang et al. (2001); *n* = 8

**Seconds**

**[16]** Claeson and Nordin (2011); *n* = 24

[17] Dalton et al. (2000); *n* = 40

[18] Frasnelli and Hummel (2003); *n* = 16

[19] Hummel et al. (2003); *n*= 24

**[20]** Kleinbeck et al. (2011); *n* = 44

[21] Mattes and DiMeglio (2001); *n* = 50

**[22]** Stuck et al. (2006); *n* = 95

[23] van Thriel et al. (2006); *n* = 144

[24] Wise et al. (2007); *n* = 26

**[25]** Ohla and Lundström (2013); *n* = 26

[26] Olofsson and Nordin (2004); *n*= 36

[27] van Thriel et al. (2008); *n* = 39

**[28]** Gui et al. (2014); *n* = 53

**Days**

[29] Kleinbeck et al. (2020); *n* = 30.

[30] Lang et al. (2008); *n* = 21

### Studies investigating ‘seconds’-exposure

#### Objective measures

Only three studies report gender differences in objective measures.

Claeson and Nordin (2011) found a significant gender effect in the detection of nasal irritation from amyl acetate.^5^ They plotted the detection proportion at seven concentrations for males and females. The exposure concentrations (1592–3849 ppm) were presented in randomized order together with interrandomized exposure to clean air. The slope of the regression differed markedly between females and males and with a steeper regression in females (cf., Fig. 1 in Claeson and Nordin 2011). This indicates that a higher percentage of males detected irritation at lower concentrations, while a lower percentage of males detected irritation at higher concentrations. The P_50_ (irritation detected by 50%) was 1597 ppm for males and 1509 ppm in females as assessed from the regression line (cf., Table 1 in Claeson and Nordin 2011). Though the detection thresholds of females and males are without statistical significance, Claeson and Nordin (2011) demonstrated that the extrapolated P_90_ (irritation detected by 90%) is about half for females (3312 ppm) than for males (6678 ppm). Consequently, while around 90% of females would indicate 3300 ppm amyl acetate as irritating less than 75% of males would do so (cf., Table 1 in Claeson and Nordin 2011). An exposure limit, however, should protect the most sensitive individuals. In this case, females appear more sensitive compared to males. However, the sample is too small and too homogenous (mean ± SD age = 25.8 ± 3.6) for generalization to the general population. Further, there might be an odor bias to report nasal irritation in response to clean air, since amyl acetate has a strong odor component (Claeson and Nordin 2011), thus, leading to concern about the presence of sensory irritation at all investigated concentrations. Furthermore, the odor component of amyl acetate might have influenced the irritation ratings more among women than men (Claeson and Nordin 2011). This is possibly due to evolutionary and hormonal explanations (Brand and Millot 2001; Claeson and Nordin 2011; Doty and Cameron 2009). Cognitive and emotional factors may play a role in gender differences (Claeson and Nordin 2011; Lundström et al. 2005; Royet et al. 2003; Thuerauf et al. 2009). Interestingly, subjective ratings of irritation intensity did not elicit a significant gender difference.

Stuck et al. (2006) showed a gender effect in eucalyptol^6^ lateralization. Thus, females demonstrated significantly higher sensitivity than males (35.64 vs. 33.00; higher values mean higher sensitivity). A gender difference was also observed in event-related potential (ERP) analyses. Amplitude and latency of the first negative peak and the second positive peak after a chemosensory event were higher in females compared to males.

It has been demonstrated that the thermal pain threshold is lower in females than in males (Gui et al. 2014). Therefore, males and females might perceive a cooling sensation of certain substances (i.e., menthol) differently leading to a proto-state of sensory irritation. Higher subjective ratings in females, hence, might be due to thermo-sensation. Furthermore, hygroscopic properties of certain substances may lead to a feeling of dryness in the eyes and nose. This perceived dryness might be a reason for ratings of sensory irritation without objective signs of sensory irritation (Dalton et al. 2018; Wolkoff 2018).

Several studies did not report significant gender differences at all, comprising nasal lateralization threshold for methyl isobutyl ketone (Dalton et al. 2000), linalool and menthol (Frasnelli and Hummel 2003), ammonia (Sundblad et al. 2004), benzaldehyde and eucalyptol (Hummel et al. 2003), ethanol (Mattes and DiMeglio 2001), formic acid, acetic acid, propionic acid, cyclohexylamine, dimethylamine, trimethylamine, ethyl formate, ethyl acetate, ethyl acrylate, methylcyclohexanone, cyclohexanone, cyclohexanol, ammonia, and hydrochloric acid (2 gender- and age-stratified samples of 72 non-smoking healthy subjects at two research institutes dividing out the substances; van Thriel et al. 2006), and temporal integration of homologous alcohols (Wise et al. 2007).

Therefore, a general higher sensitivity of females compared to males for acute sensory irritation for most substances seems unlikely based on the above studies. For a few substances, females appeared to be more sensitive; for instance, amyl acetate (Claeson and Nordin 2011) and eucalyptol (Stuck et al. 2006). Other, non-significant hints point in the same direction. However, no study showed a higher sensitivity in men.

#### Subjective ratings

Subjective ratings of irritation were higher in females compared to males in many studies. Females generally reported a significantly more intense pungency than males when exposed to nine different concentrations (ranging from odor to lateralization threshold) of six substances^7^ (formic acid, acetic acid, propionic acid, ethyl formate, ethyl acetate, and cyclohexylamine) each (van Thriel et al. 2008).

A significant gender effect was observed in ratings of nasal irritation when exposed to the highest concentration of SO_2_ 8 at 9 different concentrations (0.17 mg/m^3^ to 34 mg/m^3^; Kleinbeck et al. 2011). However, the authors argue that the dose–effect relationship of nasal irritation rating follows a saturation function that is typical for olfactory ratings, which differs from sensory irritation. However, 10% reduction of the breathing depth (tidal volume) was observed as the first objective and possible sign of sensory irritation at the highest exposure concentration. The responsible site of stimulation is not clear. It cannot be ruled out that the reduction of breathing depth is olfactory-driven. As a single breast belt was used, a change of breast breathing to abdominal breathing could be possible, though implausible in sitting subjects (Kleinbeck et al. 2011).

A gender difference in nasal irritation was not observed by exposure to menthol^9^ by nostril lateralization (Ohla and Lundström, 2013). However, the authors found gender differences in response (event-related potentials) to cineole (higher late positive component (LPC) amplitudes and different temporal patterns in females). Parameters of the EEG were analyzed, i.e., amplitudes, latencies, and more special parameters like the LPC. The LPC proved to be faster and more pronounced in females compared to males in response to cineole.

Other studies did not report gender differences at all in the perception of sensory irritation (Claeson and Nordin 2011; Olofsson and Nordin 2004; van Thriel et al. 2006).

However, as mentioned above, over longer periods (min-hours) even sub-acute concentrations can have a trigeminal impact due to temporal summation (Cain et al. 2010).

### Studies investigating ‘minutes’-exposure

Gender effects are not reported after 15 min exposure to 15 ppm acetic acid (Shusterman et al. 2005). Likewise, Yang et al. (2001) reported no gender effects in subjects’ eyes exposed for 5 min to very high concentrations of formaldehyde, a strong sensory irritant, at 1.65, 2.99, and 4.31 ppm. However, in these irritating conditions, no temporal summation took place, but an adaptation/habituation in eye blinking frequency as well as the subjective ratings. As conditions were clearly irritating (increased blinking frequency compared to clean air control condition), temporal summation could take place at lower (sub-acute) concentrations.

### Studies investigating ‘hours’-exposures

#### Objective measures

Only exposure studies with objective signs of irritation are considered. This is due to that objective sensory irritation could be questioned in many studies as markers do not increase even at the highest exposure concentration (Ernstgård et al. 2002; Gminski et al. 2011; Hey et al. 2009; Juran et al. 2012; Kleinbeck et al. 2008; Sundblad et al. 2004; van Thriel et al. 2010; Wålinder et al. 2008). However, Wålinder et al. (2008) reported a significant increase of 2.3 eye blinks/min by two-hour exposure to 10 mg/m^3^ 1-octen-3-ol,^10^ but the eye blinking frequency is still low in the exposure condition (about 8 eye blinks/minute). The change in the eye blinking frequency is probably not due to objective eye irritation but biased by the smell of 1-octen-3-ol, because the exposure concentration is below an estimated LOAEL for sensory irritation (Wolkoff 2013). Furthermore, the eye tear film was unaffected. Ernstgård et al. (2002) reported a gender effect on forced vital capacity 3 h after exposure to 50 ppm m-xylene.^11^ However, this gender difference is unlikely due to acute sensory irritation, as it could not be demonstrated immediately after exposure, and the exposure concentration is far below an estimated LOAEL (Wolkoff 2013).

Objective sensory irritation (as seen in eye blinking frequency) shows up at the end of exposure in studies with significant changes in eye irritation (4 h, 0–10 ppm peaks of ethyl acrylate, Kleinbeck et al. 2017; 4 h, 0–40 ppm ammonia, Pacharra et al. 2017, 4 h, 0–20 ppm propionic acid, Pacharra et al. 2016c; 4 h, 20 ppm 2-ethylhexanol, Schäper et al. 2015; 4 h, 0–10 ppm ethyl acrylate, Sucker et al. 2019). Though eye blinking frequencies also increase with time during the control condition (likely due to visual demands during ‘exposure’), the blinking frequencies are significantly higher at the end of the highest exposure concentration.

Most notably, objective sensory irritation from ethyl acrylate was re-investigated in a similar manner (4 h exposure; 0–10 ppm) (Kleinbeck et al. 2020, 2017; Sucker et al. 2019). A significant increase in the eye blinking frequency (e.g., 5 blinks/min in Kleinbeck et al. 2017) was seen at the highest exposure condition in comparison to the control condition (0 ppm). Neither of the studies found a gender effect on the eye blinking frequency when subjects were exposed to 5 ppm ethyl acrylate (TWA) with peaks of 10 ppm.

Other studies reported objective measures of eye irritation (eye blinking frequency), but no gender effect was observed. For instance, a change in irritation markers was observed for propionic acid (10 ppm with peaks of 20 ppm; Pacharra et al. 2016c), ammonia (20 ppm (TWA) with peaks of 40 ppm; Pacharra et al. 2017), and 2-ethylhexanol (20 ppm; Schäper et al. 2015). All exposures were at or above their estimated threshold for sensory irritation (Wolkoff 2013).

Temporal summation is usually accompanied by the respective perceptual ratings during the exposure condition (i.e., Fig. 4 in Kleinbeck et al. 2017).

#### Subjective ratings

Significant gender effects could only be observed in studies in which objective irritation is uncertain (Ernstgård et al. 2002; Wålinder et al. 2008).

Pacharra et al. (2016b) analyzed perceptual ratings in nine experiments, in which subjects were exposed to different substances (some of the experiments are described above). The results showed that trait-like modulators (i.e., odor-mediated sensitivity) affected (pungency and burning) ratings by females, but not by males.

### Studies investigating ‘days’-exposure

Kleinbeck et al. (2020) and Lang et al. (2008) exposed subjects on subsequent days to ethyl acrylate and formaldehyde, respectively. Lang et al. (2008) used five randomized sequences of 10 exposure conditions on 10 subsequent workdays for two weeks. Therefore, the sequence of exposures is unbalanced and could lead to systematic biases (e.g., systematic desensitization). Day-to-day carry-over effects cannot be excluded and could aggravate the effects of low exposures. Carry-over effects on objective and subjective markers were studied systematically by exposure of the subjects to the same concentration of ethyl acrylate for five subsequent days on objective and subjective markers (Kleinbeck et al. 2020). Carry-over effects were unobservable, neither in males nor in females from day-to-day exposure, though sensory irritation could be seen on each day.

### Summary of findings

#### Ocular irritation

Doughty (2002) reported no gender effects on spontaneous eye blinking but gender differences in the prevalence of reported eye irritation symptoms have been observed (Smith et al. 2007; van Wijk and Kolk 1997; Wolkoff et al. 2003). However, the influence of gender on sensory irritation in the eye due to experimental exposure to volatile substances could not be supported.

A large variance in eye blinking frequency may emerge over different studies due to individual differences, due to different tasks and situational factors, due to the inadequate separation of complete versus incomplete eye blinking, or insufficient data sampling for statistical analysis. Personal and occupational factors may also play a role in sensory irritation at the eye, e.g., use of contact lenses, eye make-up, certain medication, and eye diseases result in a more vulnerable tear film (Alves et al. 2023; Wolkoff 2020).

#### Nasal irritation

Gender differences only showed up in sensory irritation at the nose. It is surprising that studies that show gender differences in objective markers of sensory irritation report no gender difference in subjective markers or vice versa if both kinds of markers were measured. Differences are visible during short (seconds) exposures.

#### Irritation in the middle and lower airways

The only study that showed gender differences on objective markers (urge-to-cough; Gui et al. 2014) investigated thermal pain and not exposure to a volatile substance. However, this gender difference might explain influences other than exposure to volatile substances that might trigger or aggravate sensory irritation in females.

Generally, there is a higher incidence of chronic cough in females compared to males in the adult population regardless of exposure context (Morice et al. 2020; Morice and Kastelik 2003; Nasra and Belvisi 2009). Dicpinigaitis and coworkers demonstrated gender effects in urge-to-cough thresholds when subjects inhaled a capsaicin aerosol (non-volatile substance) with higher sensitivity in females (Dicpinigaitis et al. 2012; Dicpinigaitis and Rauf 1998). A similar effect of higher sensitivity was found in females regarding urge-to-cough with nebulized tartaric acid in a physiological saline solution (Fujimura et al. 1990). The fraction of a volatile substance reaching the lower airways is unclear due to the scrubbing effect of the nose in nasal breathing (Brüning et al. 2014; Garcia et al. 2009; Nielsen and Wolkoff 2017; Shusterman 2007). However, oral breathing together with physical exercise may increase the amount of inhaled volatile substances reaching the middle and lower airways.

In summary, no convincing evidence has been identified about a general gender difference for the endpoint ‘sensory irritation’ for most substances. However, for a few substances gender effects could be demonstrated for females, which turned out to be ‘more sensitive’ than males. This is in accordance with Nielsen and Wolkoff (Nielsen and Wolkoff 2017; Wolkoff 2013), who state that gender-related influences are, if observed at all, small. However, the influence of odor perception cannot be ruled out to bias the outcome, cf., Cometto-Muniz and Cain (1991) and van Thriel et al. (2008). Furthermore, climatic conditions (e.g., exposure to dry air) were not considered by Nielsen and Wolkoff (2017). Thus, the observed gender differences reported here may be due to the methodological shortcomings of the reviewed studies.

Nielsen and co-workers (Nielsen and Wolkoff 2017; Nielsen et al. 2007) argued that an AF of about 2 would account for the known differences in sensitivity due to gender (and age, lifestyle, and diseases). Furthermore, they claimed that a data-driven AF requires the evaluation of the effects of age, smoking, gender, and differences between eye and nasal irritation for each specific substance (Nielsen and Wolkoff 2017). Such a data-driven AF also requires consistency across studies (Nielsen and Wolkoff 2017).

In the light of possible gender effects, only six studies reported gender differences in sensory irritation due to volatile substances out of 29 reviewed studies. These have investigated a variety of different substances (See Sect. 'Characteristics of the reviewed studies', Table 7), and of which some outcomes might have been odor-driven (Claeson and Nordin 2011; Wålinder et al. 2008). However, interactions of gender and lifestyle and of gender and diseases (which both are included in the AF of 2 proposed by Nielsen and Wolkoff (2017)) are not reported in this review due to such studies are rare. Most of the reviewed studies investigate first and foremost young, healthy, non-smoking subjects. On that basis, a conservative AF of 2 for gender, age, lifestyle, and diseases is unchallenged. However, for this specific group of the general population and the respective substances, an AF for gender could be lower than 2, in part due to an odor-mediated bias. Consequently, substance-specific AFs are favorable from an economic point of view.

## Influence of grouping factor age */ older people* on sensory irritation

Table 2 shows studies comparing young adults and older adults. Further characteristics of the studies (substances, substance delivery, examined concentrations, and measures of sensory irritation) can be found in Sect. ‘Characteristics of the reviewed studies’ (Table 7). Nearly all studies investigated short exposures (seconds) in the nose. Many studies determine irritation thresholds.

**Table 2:** Studies of age effects (young vs. old adults) in sensory irritation (bold number, if significant age effects are observed)

	Exposure duration			
	Hours		Seconds	
	Objective	Subjective	Objective	Subjective
Eyes	[9]	**[9]**		
Nose		**[9]**	**[18] [19]** [20] [22] **[23] [31]**	[20] [23] [27]

**Hours**

**[9]** Schäper et al. (2015); *n* = 32

**Seconds**

**[18]** Frasnelli and Hummel (2003); *n* = 16

**[19]** Hummel et al. (2003); *n* = 24

[20] Kleinbeck et al. (2011); *n* = 44

[22] Stuck et al. (2006); *n*= 95

**[23]** van Thriel et al. (2006); *n* = 144

**[31]** Wysocki et al. (2003); *n* = 142

[27] van Thriel et al. (2008); *n* = 39

### Studies investigating ‘seconds’-exposure

Some studies showed significant age effects in objective measurements. Of these, all thresholds for sensory irritation of volatile substances in older subjects are shown in Table 3.

**Table 3:** Age differences in nasal sensory irritation thresholds

Substance	Study	Age (years)	*N*	Unit	Threshold	Sig
Menthol	Frasnelli and Hummel (2003)	18–35	8	Dilution step	1.0 in younger subjects vs. 3.3 in older subjects^a^	ANOVA: *p* = 0.001 Post hoc: menthol *p* = 0.027 linalool *p* = 0.098 (n.s.)
		59–67	8			
Linalool^b^	Frasnelli and Hummel (2003)	18–35	8		1.3 in younger subjects vs. 3.5 in older subjects	
		59–67	8			
Benzaldehyde^c^	Hummel et al. (2003)	25.4	29	Lat. Score^d^	31.2	*t*-test *p* < 0.01
		61.0	12		27.8	
Eucalyptol	Hummel et al. (2003)	25.4 (< 35)	29	Lat. Score	34.2	*t*-test *p* < 0.001
		61.0 (> 35)	12		29.8	
Propanol^e^	Shusterman et al. (2003a)	18–34	18		No numbers given according to Fig. 3b	*p* < 0.0001
		35–51	24			
		52–69	18			
Iso-amyl butyrate^f^	Stevens et al. (1982)	18–25	20	Relative sensitivity^g^	No numbers given according to Fig. 1; factor 1.6 higher in younger subjects	*t*-test *p* < 0.01
		65–83	20			
Acetic acid^h^	van Thriel et al. (2006)	18–45	19	ppm	35	ANOVA *p* < 0.05
		45-	20		46	
Cyclohexylamine^i^	van Thriel et al. (2006)	18–45	19	ppm	290	ANOVA *p* < 0.05
		45-	20		410	

^a^Low values indicate low thresholds (Frasnelli and Hummel 2003)

^b^Water solubility: slightly soluble

^c^Water solubility: slightly soluble

^d^The higher the lateralization score, the lower the threshold

^e^Miscible

^f^Water solubility: practically insoluble

^g^The higher the relative sensitivity, the lower the threshold

^h^Miscible

^i^Miscible

*n.s.* = non-significant

If there is a significant effect of age, older subjects show lower sensitivity (higher thresholds) than younger subjects.

Subjective ratings do not reflect the objective differences in thresholds (cf., Table 3). It should be considered that age-related pathologies might be responsible for the loss of nasal sensitivity (Nordin et al. 2012; Stevens et al. 1982). Further, higher odor thresholds (lower sensitivity) among older people (Olofsson et al. 2021; Sinding et al. 2014; Stevens and Cain 1987) may also contribute to the overall variance.

### Studies investigating ‘hours’-exposure

There is only one study that investigated the age effects of a longer exposure duration of 4 h with 2-ethylhexanol^12^ (Schäper et al. 2015). Sensory irritation could be demonstrated by higher eye blinking frequency at the end of the highest and constant exposure condition of 20 ppm 2-ethylhexanol (temporal summation). However, there is no difference in eye blinking frequency or in eye irritation rating between young (18–35 years) and older subjects (45–67 years). The lack of difference, however, might be due to the relatively low age of the older subjects. Age effects in trigeminally induced eye blinks by fragrances occur mostly in subjects above 60 years (cf., Acosta et al. 2006; Frasnelli and Hummel 2003; Stevens et al. 1982).

### Summary of findings

The compiled information about age effects may indicate that older people have higher thresholds. Confounding factors, i.e., (unrecognized) diseases (Rosenkranz et al. 2020), might cause higher prevalence in older age (Nordin et al. 2012). Another confounding factor could be differences in odor thresholds with lower odor sensitivity among older people (Olofsson et al. 2021; Sinding et al. 2014; Stevens and Cain 1987). On the other hand, it is well-known that older people, especially females, have a less stable eye tear film, which might result in elevated sensitivity to sensory irritants in the eyes (Wolkoff 2020).

Hence, the overall picture of possible age effects for the general population, as concluded by Nielsen and Wolkoff (2017), appears less clear, in part, since health status and other confounders may bias the outcome.

#### Age-related changes in the eye

Physiologically, there is a progressive reduction in nerve density in the human cornea occurring at the age of 70 years and older (He and Bazan 2010). This might be the effect of age-related pathologies (or eye diseases) and not a general decrease associated with age. The reduction might lead to reduced eye blinking in case of chemical exposure. Normal eye blinking has two functions: to restore the tear film and to defend the eye from environmental exposures (Alves et al. 2023; Wolkoff 2020). Therefore, eye blinking activity is essential for a healthy ocular surface; for instance, in maintaining the eye tear film stability (Wolkoff 2020). The loss of sensitivity, therefore, might result in a longer presence of a sensory irritant in the eyes.

Peshori et al. (2001) analyzed eye blinking frequency in human subjects of different ages (20–80 years). They used irritating electrical stimuli of the supraorbital branch of the trigeminal nerve to evoke blinking. The lowest stimulus intensity that reliably evoked blinking was set as threshold. For data collection, the electrical stimulus was the twofold threshold. They demonstrated a significant increase in lid-closing duration, excitability, and latency of the eye blinking in subjects over 60 years compared to younger subjects. A reduced sensitivity and higher latency of eye blinking may lead to slower clearance of the eye tear film in older adults. Therefore, a more efficient clearance may prevent sensory effects in the eyes of young adults but be detrimental to the eyes of older people with a  longer clearance time. For example, age is a risk factor for eye symptomatology (Sharma and Hindman 2014; Wolkoff 2020).

#### Age-related changes in the nose

The odor thresholds among older people are higher (lower sensitivity) than among younger people (Olofsson et al. 2021; Sinding et al. 2014; Stevens and Cain 1987). Whether sensory irritation is affected by odor perception is unclear. However, if sensory irritation ratings were (at least in part) olfactory-driven, older subjects would probably show lower sensitivity. Sunwoo et al. (2006) demonstrated that the mucociliary clearance time (removal of foreign bodies; the saccharin test), which is generally slower in older males (n = 8, mean age: 71.8 years) compared to young male subjects (n = 8; mean age: 21.7 years), is affected by 90 min exposure to low air humidity (10%) only in elderly subjects; however, the difference diminished after 180 min exposure. Ho et al. (2001) measured slower ciliary beat frequency, a higher percent of ciliary cross sections displaying single tubules, and longer mucociliary clearance time in subjects older than 40 years (41–90 years; n = 43) compared to younger subjects (11–40 years; n = 47). Consequently, harmful substances may stay longer in the nose of older people (see further discussion in Wolkoff 2018).

A detrimental effect of delayed protective reflexes in older subjects should come up with longer exposure. Consequently, older people might have higher thresholds (in ‘seconds’ exposure) indicating lower sensitivity but would need, nevertheless, lower exposure levels due to other age-related influences on the susceptibility of sensory irritation at longer exposure periods (i.e., reduced warning signs). There is a small, but demonstrable age-related decline in early warning functions of pain (Gibson and Farrell 2004). For example, Barbariga et al. (2018) found an effect of aging on nerve morphology and substance P expression (loss with age) in human corneas.

#### Age-related cognitive changes

Influence of expectations among old people cannot be ruled out. Older people might think that their eyes and nose have become less sensitive and therefore reduce their effort during threshold experiments. Miller et al. (2013) investigated the influence of priming elderly stereotypes (schematic images of a person due to group membership) on olfactory tests (Sniffin’ Sticks; Hummel et al. 1997), perceptual ratings, verbal and motor behaviors. Stereotypes were primed by describing a profound age-related decline in olfactory function in primed subjects while control subjects received general information regarding the olfactory system. They did not find an influence of priming on olfactory performance; however, verbal and motor behaviors were altered by priming older people.

## Influence of the grouping factor *age / children* on sensory irritation

### Empirical evidence

Hummel et al. (2007) found differences between age groups in the assessment of the trigeminal function (nasal lateralization) of eucalyptol in children in comparison to adults (5–54 years). Thus, lower scores (i.e., lower sensitivity) were observed in the youngest group (5 years) compared to all other, lower scores in subjects at the age of 9 compared to participants at the age of 14, and subjects at the age of 6 years had lower scores compared to’subjects aged 4 [!] and above’. However, the conclusion by Hummel et al. (2007) indicates no significant differences between subjects at age 7 and older subjects (up to 54 years). It remains unclear whether the lower score in younger children (5 years) is due to lower sensitivity or due to the test procedures used for nasal lateralization.

There is no experimental study with children younger than 5 years. Therefore, other sources of information should be used in risk assessment. One must keep in mind that impairments of childhood development may have lifelong consequences on possible effects of sensory irritation in children (Peled 2011).

### Other sources of information on sensory irritation susceptivility in children

While fetuses and children are particularly susceptible to neurotoxic effects as the brain is more sensitive during its growth stages, less is known about children’s sensitivity to sensory irritation (Berglund et al. 1992). However, developmental changes in the organs potentially affected by sensory irritation can give indirect hints.

The eye tear film stability is higher in children compared to adults (Borchman et al. 2012). Thus, the meibum of infants and children contains less CH_3_ and unsaturated C-C groups and an increased aldehyde-to-lipid hydroperoxide ratio (Borchman et al. 2012; Shrestha et al. 2011; Sledge et al. 2017; Wolkoff 2020). Further, the eye blinking frequency in infants is substantially lower (< 1 blinks/min; Mantelli et al. 2007; Zametkin et al. 1979) than in adults (Cruz et al. 2011). Furthermore, the tear break-up time is longer in infants than in adults (Cho and Yap 1993; Mohidin et al. 2002; Ozdemir and Temizdemir 2010). This indicates that infants and children have a more stable and intact eye tear film.

Berglund et al. (1992) consider children’s respiratory system as prone to the effects of indoor air pollutants that might lead to developmental impairment in the lung. Children have faster respiratory (Berglund et al. 1992; Ginsberg et al. 2005) and metabolic rates in comparison to adults (Berglund et al. 1992). However, the nasal contribution to breathing with exercise is lower in children, in part due to oral inhalation, compared to adults (Bennett et al. 2008). Therefore, a sensory irritant might reach the middle and lower respiratory system in children during exercise, while the irritant might be scrubbed in the nose of adults, depending on its water solubility (e.g., Garcia et al. 2009). Furthermore, children breathe 50% more per kg body weight compared to adults (Peled 2011). The lung epithelium, however, is not fully developed during early childhood (Foos et al. 2008; Peled 2011), while the pulmonary surface area per body weight is higher than in adults (Ginsberg et al. 2010). However, growth of the lung is not the only developmental factor (Saadeh and Klaunig 2015). All developmental changes in a child’s body may be crucial in the risk assessment process for children. For example, children’s immune system is also developing (Foos et al. 2008; Peled 2011; Schwartz 2004). All of this may cause more susceptibility in children to the systemic effects of air pollutants. This could be shown by Valcke and Krishnan (2011) for internal dose metrics in neonates compared to adults. Generalization, however, is hampered by many not well-characterized factors, e.g., the relevance of the pollutant polarity, the higher metabolic rate, and the exposed surface in the airways of children versus in the airways of adults (cf., Garcia et al. 2009).

Environmental factors like exposure to indoor and outdoor air pollutants like tobacco smoke or exhaust emissions are responsible for respiratory health effects in children (Kasznia-Kocot et al. 2010; Selgrade et al. 2008). Selgrade et al. (2008) summarized investigations on ozone (a pulmonary irritant) exposure in infant rhesus monkeys compared to clean air exposure. This showed significant changes in airway development, i.e., decreased airway size, increased number of mucous cells, and less innervation. Therefore, pharmacokinetic and pharmacodynamics age- and gender-specific models were developed (Sarangapani et al. 2003; Sweeney et al. 2015) to evaluate the risk of volatiles.

For instance, nasal modeling of airborne formaldehyde uptake (computational fluid dynamics) of human noses (2 children, 5 adults) showed that 90% of inhaled formaldehyde was extracted in the nose of adults as well as children, while a maximum of 10% may pass the nasal cavity and reach the larynx and eventually the lower airways at resting conditions (Garcia et al. 2009). Another nasal modeling showed that airborne formaldehyde-induced DNA protein cross-linking about 1.5 higher in adults than in children (Firestone et al. 2008), indicative of about 50% lower maximum effective dose in children; thus, indicative of less susceptible in children than adults for this highly water-soluble substance.

### Developmental influences

For the risk assessment of sensory irritation in children, it is reasonable to consider the development and the number of sensors as well as differences in nervous transfer.

Adverse outcome pathways represent the sequence of biological events that are caused by chemical exposures and disturb homeostasis (Frank et al. 2018). Adverse outcome pathways for sensory irritation have been described (Frank et al. 2018; Martinez and Eling 2019), and AOP key events have been defined that lead to a specific adverse outcome (OECD 2013). Such a sequence begins with MIEs, which describe initial points of interaction resulting in pertubation (OECD 2013). Frank et al. (2018) identified 5 MIEs for sensory airway irritation:

1. Activation of nociceptors
2. Peroxidation of membrane lipids
3. Induction of reactive oxygen species (ROS) and oxidative stress
4. Abrupt changes in extracellular saline
5. Phospholipase activation during detergent–membrane interactions

Each of these MIEs is directly induced by irritant exposure and can result in sensory irritation symptoms (Frank et al. 2018). Some studies show developmental aspects of these MIEs, such as nociceptors (increased physiological sensitivity to pain in neonates; Anand and Carr 1989; Bouza 2009), peroxidation of membrane lipids, and ROS and oxidative stress (Auten and Davis 2009).

For volatile substances binding to the TRPA1 receptor, Martinez and Eling (2019) defined 3 KEs leading to the adverse outcome of sensory irritation:

- (KE 1) Increase in intracellular CA^+2^ in nerves
- (KE 2) Trigeminal/vagal nerve activation
- (KE 3) Neurogenic inflammation (increase of substance P and CGRP)

#### Age-related differences in KE1 ‘Increase in intracellular CA^+2^ in nerves’

The increase in intracellular CA^+2^ is an indicator of receptor (e.g., TRPA1) activation. Banzrai et al. (2016) found developmental differences in sensory axonal excitability in normal mice using threshold tracking. Though, it is not clear whether a transfer to humans’ sensory system is possible. As an indirect hint for developmental differences in receptor activation, the density of receptors is considered as they indicate differences in sensitivity (the higher the sensor density, the higher the chance of receptor activation at a certain concentration).

In eyes, nerve terminals or free endings are responsible for transducing sensory stimuli into nerve signals (Belmonte et al. 2004; He et al. 2010). The numbers of free nerve endings are proportional to corneal sensitivity (Belmonte et al. 2004; He et al. 2010).

The sensory innervation of the cornea is derived from 30–80 stromal nerve trunks (Spadea et al. 2013; Zander and Weddell 1951). A single neuron supports 200–3000 individual corneal nerve endings (Spadea et al. 2013). Developmental aspects of corneal innervation are known for mice (He and Bazan 2016). The innervation of adult mice and adult humans share many common features (He and Bazan 2016, 2010). In mice, the innervation of the cornea still develeops after birth (He and Bazan 2016). The mouse cornea is mature after 8 weeks from birth (He and Bazan 2016). Cornea maturity age in mice corresponds to the human age of 6 years (relating average lifespans of mice and men; Dutta and Sengupta 2016). Between week 1 and week 3 the mouse cornea is mainly innervated by stromal nerves (He and Bazan 2016). Epithelial nerves were short, thin, and extended without a determined direction. Epithelial nerves derive from stromal nerve branches penetrate the subbasal layer of the cornea (He and Bazan 2016). With maturity, those nerves grow to a whorl-like structure. There is a stromal nerve regression when the mouse is mature, which might be due to neurotrophins regulating connections between nerve cells (e.g., nerve growth factor; He and Bazan 2016).

As argued above for older people, less innervation means less sensitivity of children’s eyes, which might lead to less sensory irritation but a higher susceptibility to damage as defense mechanisms (like eye blinking) may be insufficient. Contrary, the tear film stability is higher in children compared to adults (Borchman et al. 2012). Therefore, risk assessment of any volatile substance requires accounting for the complex interplay of different defense mechanisms at different developmental stages in children. It is still not known whether volatile substances might have an impact on sensory development in children at a concentration harmless to adults. Any damage to corneal nerves, however, leads to diminished corneal sensitivity and could lead to long-term alterations in the functional integrity of the ocular surface (Marfurt 2010; Marfurt et al. 2010; Medeiros and Santhiago 2020; Spadea et al. 2013). Although corneal nerves are capable of regeneration, this process is slow and imperfect (Spadea et al. 2013). For example, regeneration after most corneal surgeries is accompanied by reduced nerve density, alterations in nerve architecture, and consequently lower corneal sensitivity (Spadea et al. 2013). In children, any damage to sensory nerves might lead to long-term impairment that must be considered in risk assessment.

#### Age-related differences in KE2 ‘Trigeminal/vagal nerve activation’

In addition to differences in nerve density, there might be differences in nerve transmission during developmental changes in children. Uppal et al. (2016) investigated the neural dynamics of somatosensory processing of adaptation across childhood via vibrotactile stimuli at the children’s wrist. Differences in somatosensory evoked potentials could be identified in different age groups (6–10, 10–13, and 13–17 years old). The authors conclude that reactivity to tactile stimulation seems to change dramatically during childhood. Uppal et al. (2016) speculate that such difference might be due to the co-working of’immature’ and’mature’ networks; for instance, there could be different configurations of neural generators in somatosensory cortices or developmental changes in the anatomy. Otherwise, it is possible that skull thickness might influence the scalp-reported signal, though the underlying neuronal response was the same (Uppal et al. 2016).

Many populations of neurons in the vertebrate nervous system pass through 4 phases of development (Davies 1988):

1. Differentiation from progenitor cells
2. Growth of axons to their target fields
3. Target field innervation
4. Remodelling axon connections

Though much of the trigeminal system of rats develops during the embryonic stage (Davies 1988; Stainier and Gilbert 1991) there is also postnatal development of the trigeminal system (Toma et al. 2006). For example, glial elements of root entry zones in the central nervous system are mainly developed postnatally in rats (Toma et al. 2006) until the end of week 2 (corresponding to 1 year in humans, relating average life spans of rats and men; Andreollo et al. 2012). Therefore, the central nervous system parts of mammalian trigeminal systems seem not to be mature at birth. This could lead to stronger adverse perceptions in children at a certain developmental stage exposed to stimulus intensities, which would not be adverse in children at other developmental stages (cf., vibrotactile stimuli in Uppal et al. 2016).

#### Age-related differences in KE3’Neurogenic inflammation (increase of Substance P and CGRP)’

Distribution of the neural peptides substance P and calcitonin gene-related protein (CGRP) was determined by staining in mice cornea (He and Bazan 2016) with a higher content of CGRP than that of substance P in mice epithelial innervation. However, the authors did not investigate developmental changes in this distribution. In older studies, the pattern of corneal CGRP innervation of rats continuously changes during the first 3 weeks after birth (1.5 years in humans, relating average life spans; Andreollo et al. 2012) and then reaches the adult state (Jones and Marfurt 1991). During week 1, for example, CGRP innervation density of the pericentral corneal epithelium increases relative to the peripheral corneal epithelium, intraepithelial axons become more branched, thinner, more prominently beaded, and the number of free nerve endings increases (Jones and Marfurt 1991). This suggests that less CGRP might be released due to sensory irritation of the eye in children compared to adults due to lower availability. The same might be true for substance P as 99% of all corneal protein gene product (PGP)-p.5-immunoreactive nerves contain both CGRP and substance P in dog corneas (Marfurt et al. 2001).

Risk assessment for children, therefore, requires consideration of developmental changes affecting MIEs and KEs.

### Summary of findings

The only experimental study with children demonstrated that 7-year-old children are as sensitive to nasal irritation as adolescents and adults (Hummel et al. 2007). Therefore, some researchers consider that risk assessment of sensory irritation in children might not be of special concern (Dourson et al. 2010). Others conclude that children’s risk assessment requires special consideration due to developmental changes (Saadeh and Klaunig 2015). For example, Hunter et al. (2010) identified postnatal phases of special susceptibility to ozone in rats; however, ozone is not a sensory, but a pulmonary irritant.

Saadeh and Klaunig (2015) conceptualize different risk factors for children, inter alia physiological and immunological development, breathing rate, physical activity, and respiratory system development. They integrate the available methods for children’s inhalational risk assessment by comprehensive PK/PD modeling of different target sites. Such modeling could be used to identify target sites at which children might be more sensitive or obtain a higher dosage compared to young adults. A quantification of differences (if identified) must be the next step.

For occupational risk assessment, Maier et al. (2014) argue that young naïve non-smoking adults represent the most responsive sub-group for sensory irritation in controlled exposure studies. Regarding the argumentation above, this claim cannot be transferred to the general population. Children and older people might suffer more from the same exposure to sensory irritants than young naïve adults. Furthermore, certain (chronic) diseases might influence the sensitivity (even in naïve non-smoking young adults).

## Influence of the grouping factor *health status* on sensory irritation

Different diseases have an impact on the eyes and the airways. For example, inflammatory diseases have a direct impact on the sensors involved in sensory irritation. Furthermore, acute impacts can lead to worsening (exacerbation) of chronic lung diseases (‘acute on chronic’ lung disease; Kreuter and Cottin 2017). Environmental pollution and occupational exposures elicit such exacerbations, for example in asthma and COPD (Eisner et al. 2010; Vogelmeier et al. 2017; Skaaby et al. 2021).

### Allergy/asthma

In respiratory allergies, substances that are harmless to the organism can elicit an immune response. The first step in developing a respiratory allergy is the sensitization to a substance (called antigen; Briatico-Vangosa et al. 1994). Such sensitization can be classified into 4 hypersensitivity reactions (Dispenza 2019; Gell and Coombs 1963). The most common type which incorporates hay fever and allergic asthma (cf., prevalence in Germany, Bergmann et al. 2016) is Type 1 allergy. The critical event during sensitization is the development of mast cell binding antibodies (immunoglobulin E; IgE). An individual is sensitized if during an immune response antigen-specific IgE antibodies are produced that ‘prime’ mast cells in various tissues (Briatico-Vangosa et al. 1994). Consequently, repeated exposure to the same or similar substance leads to a hypersensitivity reaction (Briatico-Vangosa et al. 1994). The neuropeptide nerve growth factor is a neurotrophic factor that plays a critical role in hypersensitivity (Päth et al. 2002). A sensitized individual might develop an allergy on subsequent exposure to the antigen. Activated mast cells, other inflammatory cells, and resident cells can stimulate nerve endings and cause long-lasting changes in neuronal excitability (Undem and Taylor-Clark 2014). Such a reaction is an exaggerated response of the sensory system due to interaction with sensory nerves, changes in central nervous processing, and altered transmission in sympathetic, parasympathetic, and enteric autonomic nerves (Undem and Taylor-Clark 2014).

Seasonal allergic rhinitis and asthma are inflammatory diseases affecting the airways, first the upper airways and second the lower respiratory tract. Both are linked by a common pathogenic process with overlapping inflammatory cells, mediators, and cytokines (Bjermer 2007). The bridge between the upper and lower airways seems to be systemic inflammation (Alving and Malinovschi 2010; Bjermer 2007), which might lead to a higher sensitivity against sensory irritants. Therefore, studies on elevated sensitivity in seasonal allergic rhinitis and in asthma were analyzed together.

Furthermore, exposure to some volatile substances by itself might generate an allergic disease (Nurmatov et al. 2015; Tagiyeva and Sheikh 2014), i.e., occupational asthma (Maestrelli et al. 2009) or Reactive Airway Disease Syndrome (RADS; Johnson et al. 2019). Some volatile substances activate the TRPA1 receptor by forming a Michael addition product with a cysteine residue of TRPA1 through covalent protein modification; thus, as a consequence, causing allergic reactions (i.e., isothiocyanate and sulfides; Mihara and Shibamoto 2015).

Many studies have experimentally investigated differences between subjects with allergies and healthy control subjects. All of these have in common that there is a self–selection of participants, i.e., only subjects with mild (or moderate) allergy may participate. In other words, people with severe disease and assumed higher sensitivity probably deselect such exposure studies. Table 4 shows the studies investigating allergy/asthma effects in sensory irritation. Further characteristics of the studies (substances, substance delivery, examined concentrations, and measures of sensory irritation) can be found in Sect. ‘Characteristics of the reviewed studies’ (Table 7).

**Table 4:** Studies of allergy effects (non-allergic vs. allergic subjects) in sensory irritation (bold number, if significant allergy effects is observed)

	Exposure duration					
	Hours		Minutes		Seconds	
	Objective	Subjective	Objective	Subjective	Objective	Subjective
Eyes	[7] [10] [12]	[7] [10] [12] **[32]**			[36]	[36]
Nose	[7] [10] [12]	[7] [10] [12] **[32]**	**[14]** [33] [34]		**[37]**^**b**^ [36]	**[37]**^**b**^ [36]
Pharynx/Larynx; Throat		**[32]**	**[35]**^**a**^	**[35]**^**a**^	[36]	[36]
Trachea/Bronchi			**[35]**^**a**^	**[35]**^**a**^	[36]	

^a^Stimulus is hot air

^b^Stimulus is mannitol powder

**Hours**

[7] Pacharra et al. (2017); *n*= 37

[10] Sucker et al. (2019); *n* = 22

[12] Wålinder et al. (2008); *n* = 29

**[32]** Fadeyi et al. (2015); *n* = 71

**Minutes**

**[14]** Shusterman et al. (2005); *n* = 16

[33] Shusterman et al. (2003b); *n* = 52

[34] Shusterman et al. (1998); *n* = 16

**[35]** Khosravi et al. (2014); *n* = 13

**Seconds**

[36] Petrova et al. (2008); *n* = 40

**[37]** Koskela et al. (2000); *n* = 30

#### Studies investigating ‘seconds’-exposure

Shusterman et al. (2003a) demonstrated that rhinitis status could predict VOC localization of n-propanol (indicator of irritation); allergic rhinitis sufferers turned out to be more sensitive than the healthy controls (no numbers given). An odor-driven bias reaction cannot be ruled out.

It was demonstrated that markers of sensory irritation depended on the severity of rhinitis by comparison of exposed subjects with non-active and active rhinitis with healthy controls to mannitol (dry powder mannitol; 200 mg mannitol/mL; Koskela et al. 2000). An increase in 15-hydroxyeicosatetraenoic acid (molar mass = 320.5; an index of epithelial cell activation) correlated with nasal symptoms for itching and burning. Nasal symptoms increased even until 10 min after the challenge in subjects with active allergic rhinitis, while this was not the case in healthy subjects. Furthermore, nasal peak inspiratory flow was reduced after the mannitol challenge in patients with active allergic rhinitis. However, the transfer of these results to volatile substances is not possible.

Petrova et al. (2008) could not identify an elevated sensitivity (ocular threshold, nasal threshold, combined threshold) to ammonia in mild to moderate asthmatics compared to healthy control subjects. However, none of the asthmatics had acute inflammatory or rhinitis symptoms during the test sessions.

#### Studies investigating ‘minutes’ exposure

Shusterman et al. (2005) exposed subjects with and without seasonal allergic rhinitis to acetic acid (15 ppm) for 15 min. The subjects with rhinitis were tested outside their relevant pollen season. The nasal airway resistance compared to baseline was greater among the subjects with rhinitis compared to those without rhinitis immediately and 15 min after the exposure.

Shusterman et al. (1998, 2003b) exposed subjects with and without seasonal allergic rhinitis to chlorine^13^ (1 ppm; a reactive substance) for 15 min. The nasal airway resistance compared to baseline was greater in the rhinitis group immediately and 15 min after exposure compared to the group without rhinitis. However, the subjective rating of nasal irritation was low (closer to the label ‘none’ than to ‘slight’) so that sensory irritation might not be the reason for this physiological reaction. An odor-mediated bias for both studies cannot be ruled out.

#### Studies investigating ‘hours’ exposure

Pacharra et al. (2017) describe weak changes in the eye blinking frequency (marker of sensory irritation) by exposure to ammonia (peaks of 40 ppm, 20 ppm TWA). Subjects with seasonal allergic rhinitis did not show a higher sensitivity than healthy control subjects, neither in subjective ratings nor in objective markers of sensory irritation. The study was conducted off-season, subjects with seasonal allergic rhinitis still showed higher fractional exhaled NO (FeNO). There might be a self-selection of subjects with mild allergic rhinitis since those with moderate or severe symptoms are likely to deselect participation in an exposure study.

Sucker et al. (2019) found differences in perceptual ratings only during sham exposure to ethyl acrylate^14^ with higher ratings in atopic subjects compared to healthy control subjects. Moreover, the interaction between atopy and ethyl acrylate exposure was not significant. However, they observed that the eye blinking frequency in all atopics was higher than 20 blinks/min, while the healthy control subjects showed 8 to 37 blinks/min. Yet, the main effect of atopy on eye blinking was only marginal.

Fadeyi et al. (2015) exposed asthmatic and non-asthmatic subjects to a mixture of ozone-limonene reaction products (i.e., formaldehyde^15^) in a field environmental chamber for 3 h. Odor intensity and sensory irritation were rated lower by asthmatic subjects compared to healthy control subjects. Perceived physiological–like symptom ratings (flu, chest tightness, and headache) of asthmatics, however, were often higher compared to the non-asthmatic subjects. It is unclear whether sensory irritation took place as objective markers of sensory irritation were not measured. Although the report of sensory irritation is low among both groups of subjects, the finding is compatible with the fact that asthmatics produce more mucous acting as a scrubber (e.g., Garcia et al. 2009), in agreement with the finding that sensitized mice are less sensitive than normal mice to formaldehyde at low relative humidity (Larsen et al. 2013). In general, no convincing evidence has been found that asthmatics are more sensitive than healthy subjects to formaldehyde (a highly water-soluble substance), as reviewed by Wolkoff and Nielsen (2010); a conclusion later supported by Golden (2011).

#### Summary of findings

There is inconclusive evidence of higher sensitivity in subjects with allergic diseases. Subjects with seasonal allergic rhinitis were usually investigated outside the pollen season (Pacharra et al. 2017; Shusterman et al. 2005; Sucker et al. 2019). It remains unclear whether the described (lack of) effects could also be seen during pollen season with acute complaints and whether a medication during pollen season has additional effects. Still, a minimal persistent inflammation can also be observed in patients with seasonal allergic rhinitis outside the pollen season (Ricca et al. 2000).

As mentioned by Pacharra et al. (2017) and above, individuals with a severe allergic disease probably deselect participation in such exposure studies. Therefore, other sources of knowledge must be considered.

There are indirect hints that airway inflammation (due to acute allergy) might change the sensitivity to sensory irritants based on animal experiments and knowledge about inflammatory processes during allergic phases in the eyes and airways. For instance, Belvisi (2003) proposed that changes in Aδ- und C-fibers in airway inflammation might lead to exaggerated function in response to the inflammatory process. The next paragraphs discuss such influences.

##### Potential influences of allergic diseases on the eye

Acosta et al. (2013) investigated keratoconjunctivitis in guinea pigs. They recorded changes in the electric activity of cornea conjunctival sensory nerve fibers in response to CO_2_ following an ocular allergic challenge provoked by ovalbumin. After repeated exposure to the allergen, the frequency of impulses in polymodal fibers was significantly higher and the impulse latencies were shorter compared to controls. The response to heat, however, was lower after the first allergic challenge but returned to control levels after the repetitive exposures.

##### Potential influences of allergic diseases on nose/airways

Opiekun et al. (2003) could not identify differences in objective markers of sensory irritation (ocular redness, nasal mucosal swelling) when exposing asthmatics and healthy control subjects to fragrances, though asthmatics reported more nasal symptoms. No sensory irritation could be provoked by the fragrances at the investigated concentrations.

Asthma is frequently cited as an outcome of adverse health events relating to air fresheners (Johnson et al. 2019). Fragrances inhaled in indoor air are usually below thresholds for sensory irritation (Nielsen and Wolkoff 2017). However, asthma symptoms can be triggered by very low doses of the sensitizer and irritants can exacerbate existing asthma (Johnson et al. 2019). Basketter et al. (2019) reviewed the impact of fragrances on asthmatics compared to healthy subjects. They conclude that adverse health effects caused by inhalation of fragrances are uncommon, and a convincing biological and mechanistic basis is lacking.

Volatile substances might cause respiratory allergies (i.e., occupational asthma; Baur et al. 2012; Briatico-Vangosa et al. 1994). Several substances have been identified that can cause hypersensitivity when inhaled (Baur et al. 2012; Briatico-Vangosa et al. 1994; Nurmatov et al. 2015). Work-related asthma can be divided into occupational asthma (new onset asthma) and work-aggravated asthma (Baur et al. 2012). Occupational asthma is elicited by occupational allergens with well-defined etiological role and IgE-mediated patho-mechanisms as well as occupational agents with unknown patho-mechanism (Baur et al. 2012). Atopics with nonspecific bronchial hyper-responsiveness are a susceptible group that is also affected by chronic exposure to relatively low doses of irritant gases, fumes, or aerosols (Baur et al. 2012; Burge et al. 2012; Dykewicz 2009; Kipen et al. 1994). Often, such chronic causative concentrations of irritants are below their OELs or permissible exposure limits (Baur et al. 2012).

Subjects suffering from allergic rhinitis show a greater increase in nasal resistance than healthy subjects when they were exposed to substance P and methacholine (Devillier et al. 1988). This effect might be due to the increased number of basophils and mast cells in and on the nasal epithelium of the allergic patients (Devillier et al. 1988; Nielsen 1991) indicating a higher vegetative reaction capacity.

Oetjen and Kim (2018) describe the reciprocal interactions between the immune and the sensory nervous system. The immune system shapes the scope and intensity of sensory responses by modulating the sensory nervous system. For example, the TRP channels can be activated by volatile substances but also act as a common pathway for many different immune-cell-derived stimuli (Chiu et al. 2012; Oetjen and Kim 2018). These proteins encompass different channels that regulate different sensory responses like nociception and thermo-reception (Bautista et al. 2014; Oetjen and Kim 2018). For example, chronic inflammation can affect neuronal physiology by increasing innervation at sites of inflammation (Jia and Lee 2007; Oetjen and Kim 2018). O'Hanlon et al. (2007) could demonstrate that nerve fibers in the epithelium, subepithelium, and glandular/vascular regions are significantly increased in allergic rhinitis. Nasal sensitivity was correlated with protein gene product 9.5 subepithelial innervation as well in subjects with allergic rhinitis as in healthy subjects (O’Hanlon et al. 2007). Furthermore, Undem et al. (2000) describe changes in neural activity in allergic diseases, thus, allergic inflammation affects inter alia the primary afferent nerve leading to changes in neuronal excitability. Allergic inflammation may cause central sensitization, which might modulate neural reflexes (Undem et al. 2000).

It is hypothesized that even skin allergy might result in higher respiratory responsiveness (Arts et al. 2006b) indicating that skin and respiratory allergy are not separate diseases in animal models (Arakawa et al. 1995; Arts et al. 2006b, 2004, 1998; Blaikie et al. 1995; Botham et al. 1989). However, in humans, contact sensitization of a fragrance mix leads to a higher rate of bronchial hyper-responsiveness in women (Schnabel et al. 2010) and of non-allergic rhinitis (Gelardi et al. 2017). Furthermore, inhalation of fragrances may heighten the risk of developing contact dermatitis in patients with contact allergy (Schnuch et al. 2010). A sensitization of the respiratory tract, therefore, can be facilitated by the skin and inhalative exposure (Kimber et al. 2018). Many volatile substances can migrate across the skin and trigger immune responses (Kimber et al. 2018). A hybrid (combined) AOP for both skin and respiratory sensitization is proposed comprising 4 KEs considering communalities and differences between contact allergens and chemical respiratory allergens (cf., Fig. 2 in Kimber et al. 2018). This hybrid AOP highlights where the pathways of skin sensitization and sensitization of the respiratory tract overlap and where they may diverge.

##### Allergy in rats and mice

Fujimaki et al. (2004) showed that exposure to formaldehyde (40; 80; 2000 ppb for 12 weeks) can induce differential immunogenic and neurogenic inflammatory responses (Interleukin 1, nerve growth factor, interferon-γ) in an allergic mouse model at different exposure concentrations in comparison to non-allergic mice. The results should be assessed carefully, because the highest level of formaldehyde would have induced stress-related reactions, because of a substantial reduction of the breathing rate in the mice, according to Nielsen et al. (1999).

Morris et al. (2003) investigated breathing responses to irritants in the healthy and allergic airway-diseased mice. Mice with ovalbumin-induced allergic airway disease showed enhanced breathing patterns and/or obstructive responses.

However, Hansen et al. (2016) investigating mice with airway allergy observed no aggravation of allergic symptoms when exposed to ozone or a mixture of ozone and limonene reaction products. They even hypothesized that anti-inflammatory properties of the tested limonene-containing pollutants might have attenuated airway allergy (Hansen et al. 2016).

Neither air humidity nor allergic sensitization (by ovalbumin) had an impact on sensory irritation by 5 ppm formaldehyde in mice (Larsen et al. 2013). This might be due to a more efficient scrubber effect for this highly water-soluble substance caused by increased mucus production in asthmatic animals and in agreement with Garcia et al. (2009).

However, it is undisputed that capsaicin-sensitive sensory nerve mechanisms are upregulated by allergy-caused sensitization and inflammation in the airways (Lundberg 1995; Saria 1988).

### Chronic obstructive pulmonary disease (COPD).

Chronic obstructive pulmonary disease (COPD) is ‘a disease state with progressive airflow obstruction.’ (Bose et al. 2015 p. 245). COPD leads to epithelial cell damage, bronchoconstriction, lung parenchymal destruction, and mucus hypersecretion (Bose et al. 2015). There are TRP channels in the lung epithelium that are activated by irritants like chlorine (reactive substance) or aldehydes, which could contribute to COPD exacerbation (Bose et al. 2015). Bose et al. (2015) reviewed the role of oxidative stress and TRP channels in COPD. Inhalation of substances, triggering the redox-sensitive signaling cascades, can cause cough, wheezing, and the exacerbation of airway inflammation, especially in susceptible people with asthma and COPD, children and older people (Zholos 2015). Oxidative stress leads to lipid peroxidation which might result in an inflammatory response (Zholos 2015). Several TRP channels are capable of sensing reactive oxygen species and reactive nitrogen species (Shimizu et al. 2014), i.e., TRPM8 in COPD.

Basoglu et al. (2015) investigated the influence of aerosolized Adenosine 5’-triphospate (ATP) in smokers and patients with COPD compared to non-smoking controls. COPD patients reported the strongest change in dyspnea on a Borg scale (change between pre- and post-provocation with ATP) compared to smokers (lower change) and healthy controls (no change). Because of the inflammatory process, significant amounts of ATP are released from various cells, inter alia by nerve endings (Basoglu et al. 2015). Therefore, the inflammatory response of sensory irritation might aggravate COPD by releasing ATP to extracellular space.

Chronic cough is a symptom of COPD (Nasra and Belvisi 2009). The cough reflex is usually initiated by the activation of airway sensory nerves, i.e., C-fibers and Aδ-fibers (Nasra and Belvisi 2009). However, ‘cough receptors’ do not express TRPV1 receptors or substance P (Mazzone 2004; Ricco et al. 1996). Nevertheless, a lowered threshold to aerosolized citric acid- and capsaicin-induced (non-volatile substances) cough has been demonstrated in patients with asthma or COPD (Doherty et al. 2000; Gatti et al. 2006; Higenbottam 2002; Wong and Morice 1999).

Belvisi et al. (2011) summarize that the activation of the TRPA1 receptor on vagal sensory afferents in the lung by irritant substances could lead to central reflexes (dyspnea, changes in breathing pattern, and cough) which might aggravate symptoms and pathophysiology of respiratory diseases.

Gatti et al. (2006) investigated the mechanism by which chronic inflammation (like in asthma or COPD) leads to a lowered cough threshold in Guinea pigs. They found that protease-activated receptor-2 stimulation exaggerates TRPV1-dependent cough by different mechanisms.

Shapiro et al. (2021) showed that the airway length and the number of branch points are significantly increased in chronic cough compared to healthy subjects in the human epithelium. Influence of age and gender was not found.

### Impact of other diseases on the neurosensory system

Benoliel et al. (2006) found changes in the trigeminal neurosensory system following acute and chronic paranasal sinusitis in human subjects. They measured electrical detection thresholds for large myelinated nerve fibers and heat detection thresholds for thin unmyelinated nerve fibers. They observed hypersensitivity of large myelinated nerve fibers in early inflammatory neuritis, while long-lasting processes may result in hyposensitivity as they are, presumably, accompanied by nerve damage (Benoliel et al. 2006).

Auais et al. (2003) observed immunomodulatory effects of sensory nerves in rats during respiratory virus infection. The stimulation with capsaicin led to the recruitment of numerous lymphocytes (CD4 and T-cells) and monocytes in infected rats, while this could not be observed in healthy rats. Virus infection induced overexpression of NK1-receptors for substance P on lymphocyte subpopulations in the lymphoid tissue (Auais et al. 2003).

Eye tear dysfunction is generally associated with tear film instability, hyperosmolarity, and corneal sensitivity leading to eye-irritating symptoms in human subjects (Rahman et al. 2015). Other ocular diseases caused by environmental and occupational conditions further aggravate the tear film stability, which might lead to elevated vulnerability to external stimuli (Wolkoff 2020).

### Summary of findings

There is ample evidence that the sensory system responsible for sensory irritation evoked by volatile substances is affected in airway diseases (i.e., TRPV1; Szallasi 2002). Bessac and Jordt (2008) argue that both TRPV1 and TRPA1 sensors, which play a major role in sensory irritation caused by volatile irritants, are also important in airway reflex control. Therefore, they may contribute to chemical hypersensitivity, chronic cough, airway inflammation in asthma, COPD, and reactive airway dysfunction syndrome. TRPV1 and TRPA1 (together with TRPV4 and TRPM8 receptors) are affected by airway diseases (Grace et al. 2014). Thus, there is a complex interplay in diseases, where the sensory system is affected and contributes to diseases in the airways. The same may be true for the sensory system of the eyes.

The quantification of differences between healthy individuals and individuals with diseases is difficult. Johansson et al. (2016) suggested a three-fold higher sensitivity of asthmatics compared to healthy individuals. Even a nine-fold difference in sensitivity was identified for sulfur dioxide. However, this study’s outcome should be considered cautiously because substantial differences between asthmatics and healthy people were only identified for a few substances. Furthermore, the differences could be ascribed to oral inhalation breathing or the substances were pulmonary irritants or both sensory and pulmonary irritants, as for sulfur dioxide, see discussion in Nielsen and Wolkoff (2017). The study does not support a general AF for asthmatics, rather, protection of asthmatics should be based on a substance-by-substance approach.

Furthermore, the perception of sensory irritation depends on personal factors, which are discussed in the next paragraph.

## Influence of the grouping factor *vulnerability* on sensory irritation

Individuals might differ in the amount of ‘adversity’ of perceived sensory irritation. Differences arise from differences in the sensory system itself or from differences in the evaluation of the perception delivered by the sensory and olfactory systems. For example, expectations and beliefs (i.e., health risk perception; Dalton 1999), reactions of co-workers and bystanders, and affective orientation (Watson et al. 1988) influence the perception and report of irritation. Perceived health risk is also able to modulate inflammatory airway response in asthmatics (Jaén and Dalton 2014). Psychosocial factors like personality and stress might lead to building-related complaints and could even exacerbate CI and symptoms (Dalton and Jaén, 2010). External factors may alter the perception of volatile substances. Furthermore, there is a high individual variation in reporting trigeminal sensitivity; consequently, what is irritant to one might barely be noticed by another subject (Dalton and Jaén, 2010). Therefore, exposure to odors and irritants can lead to adverse responses in (vulnerable) people with MCS, which is also known as idiopathic environmental intolerance (Dalton and Jaén, 2010). As there are no uniform diagnostic criteria for MCS (Eis et al. 2008), declarations of prevalence range from 0.5% to 33% (Dantoft et al. 2021; Rossi and Pitidis 2017; Zucco and Doty 2022; highest prevalence in studies without a medical evaluation).

Multiple chemical sensitivity is a syndrome of non-specific multisystem symptoms caused by exposure to common odorous substances at non-toxic levels (Azuma et al. 2015; Cullen 1987; Dalton and Jaén, 2010; Eis et al. 2008; Graveling et al. 1999; Nordin 2020; Rossi and Pitidis 2017; Winder 2002). Exaggerated responses in MCS subjects might be caused by differences in the sensory system or in the evaluation of the perception (Nordin 2020). Neurogenic inflammation can be the result of volatile substances interpreted as threats that act as stressors upon the body (Nordin 2020). Consequently, the threshold for sensory perception in MCS subjects might be lower (Nordin 2020).

Studies investigating the impact of volatile irritants on sensory irritation in MCS patients are shown in Table 5 and associated substances are listed in Sect. 'Characteristics of the reviewed studies' (Table 7). MCS patients were identified by criteria developed by Cullen (1987), while other studies rely on self-reporting using questionnaires (self-reported MCS, sMCS).

**Table 5:** Studies of sensory irritation in MCS and healthy subjects (bold number, if significant differences are observed)

	Exposure duration					
	Hours		Minutes		Seconds	
	Objective	Subjective	Objective	Subjective	Objective	Subjective
Eyes	[38]	**[39] [38] [40]**		[44] [45]		
Nose	[41]	**[39] [40] [41]**	[42] [43]	[42] [44] [45]	[46]	**[47] [48] [46] [27]**
Pharynx/Larynx; Throat			[42]			
Trachea/Bronchi			[42]			

**Hours**

**[38]** Kiesswetter et al. (2005) / van Thriel et al. (2005); *n* = 24

**[39]** van Thriel et al. (2002); *n* = 24

**[40]** Seeber et al. (2002); *n* = 160

**[41]** Pacharra et al. (2016b); v = 26

**Minutes**

[42] Andersson et al. (2015); *n* = 36

[43] Dantoft et al. (2015); *n*= 36

[44] Claeson and Andersson (2017); *n* = 37

[45] Österberg et al. (2004); *n* = 100

**Seconds**

**[27]** van Thriel et al. (2008); *n* = 39

**[46]** Nordin et al. (2005); *n* = 38

**[47]** Azuma et al. (2016); *n* = 16

**[48]** Alobid et al. (2014); *n* = 118

### Studies investigating ‘seconds’-exposure

#### Objective markers

Nordin et al. (2005) investigated chemosensory ERPs in self-reported hyper- and hyposensitive subjects (as measured by the Chemical Sensitivity Scale; Nordin et al. 2003). Ratings of malodorous pyridine^16^ exposure were higher in hypersensitive subjects compared to hyposensitive subjects. However, this effect is not reflected in ERP amplitudes or latencies (no group differences; Nordin et al. 2005). The authors argue that the neural cortical basis for this effect might be an uncaptured higher cortical level not acquired by chemosensory ERPs.

#### Subjective ratings

Female subjects with MCS rated the irritation of the odorant γ-undecalatone^17^ (no concentration given) higher than female subjects without MCS (Azuma et al. 2016). MCS was diagnosed according to the 1999 consensus criteria (Anonymus 1999). However, there is no indication of any sensory irritation beyond subjective ratings.

In another study, females with MCS rated irritation caused by odorants higher than females without MCS; likewise, the odor was perceived more intense (Alobid et al. 2014).

van Thriel et al. (2008) did a median-split on sMCS rating (based on the chemical and general environmental sensitivity questionnaire; Kiesswetter et al. 1999) on their subjects without a preselection. Nevertheless, when exposed to six different volatile substances^18^ at nine different concentrations (ranging from odor threshold to nasal irritation threshold) for several seconds (flow olfactometry), subjects with higher sMCS values rated pungency more intense (p = 0.06, n.s.).

### Studies investigating ‘minutes’-exposure

#### Objective markers

Andersson et al. (2015) investigated the breathing rate of 18 MCS subjects (2 males) in response to a low concentration of n-butanol^19^ (3.7 ppm) for 50 min compared to subjects without MCS. MCS was diagnosed according to criteria described by Lacour et al. (2005). No differences in breathing parameters were observed, though subjective ratings of odor intensity and symptoms were higher in the MCS group (even during ‘sham exposure’).

Dantoft et al. (2015) investigated the epithelial lining fluid from the nasal cavity of MCS and healthy control subjects before, during, and after exposure to 3.7 ppm n-butanol for 50 min (different aspect but same experiment as described in Andersson et al. 2015). No abnormal upper airway inflammatory mediator levels in 19 cytokines and chemokines could be observed in the MCS subjects (Dantoft et al. 2015), i.e., no indication of sensory irritation. It should be noted, however, that the n-butanol concentration of 3.7 ppm is a factor of about 50 below an estimated LOAEL for sensory irritation (Wolkoff 2013), and a factor of 100 above its P_100_ odor threshold (Nagata, 2003).

#### Subjective ratings

Claeson and Andersson (2017) exposed subjects with or without CI to heptane in combination with 70 µg/m^3^ acrolein,^20^ a strong sensory irritant. Chemical intolerance was addressed by a single question. Compared to control subjects, subjects with CI reported greater sensory irritation in the eyes, nose, and throat when exposed to heptane-masked acrolein at a concentration considered to be below the sensory irritation threshold. It remains unclear whether heptane has masked the smell of acrolein completely. If not, this would lead to different odor hedonics in the two experimental conditions (heptane vs. heptane + acrolein) that could influence the reported sensory irritation. Furthermore, it is uncertain whether the acrolein concentration is above the threshold for sensory irritation, cf., Dwivedi et al. (2015), Wolkoff (2013).

### Studies investigating ‘hours’-exposure

#### Objective markers

Kiesswetter et al. (2005) investigated eye blinking frequencies in sMCS subjects and control subjects during exposure to 2-ethylhexanol (20 ppm). The eye blinking frequencies of sMCS subjects (identified according to Kiesswetter et al. 1999) did not differ significantly from those of control subjects. No difference was observed between female subjects with generalized self-reported CI (defined by questionnaires, cf., Pacharra et al. 2016a) and female control subjects in nasal inflammatory markers (substance P, TNF-α) after exposure to ascending concentrations steps up to 10 ppm ammonia, a concentration well below the lateralization threshold (Pacharra et al. 2016d; Smeets et al. 2007) for 75 min. The addition of subjects’ olfactory function (measured by Sniffin’ Sticks; Hummel et al. 1997) as a covariate resulted in higher TNF-α in the CI group. However, this effect was not modulated by exposure (pre-post-comparison; Pacharra et al. 2016a).

#### Subjective ratings

Van Thriel et al. (2005) re-analyzed the subjective ratings of the above study (Kiesswetter et al. 2005). Only ratings of eye irritation symptoms (Swedish Performance Evaluation System, SPES; Iregren 1998)^21^ were higher during constant exposure in the sMCS group compared to the control subjects.

sMCS subjects reported higher ratings of eye and nose irritation (measured by SPES) than healthy controls when exposed to 2-butanone^22^ and ethyl benzene^23^ for 4 h not exceeding their OELs at that time (varying concentrations at an average of 189 ppm 2-butanone and 98 ppm ethyl benzene) (van Thriel et al. 2002). While the ratings of sMCS subjects increased over time, the ratings of control subjects stayed the same at a low and constant level of 10 ppm butanone and 10 ppm ethyl benzene.

Seeber et al. (2002) carried out and analyzed 14 experimental inhalation studies with mostly 4 h’ exposures to different volatile substances^24^ at constant or changing concentrations. Eye and nose irritation was rated by the subjects, some with sMCS. Mean ratings of eye and nose irritation were higher in sMCS subjects than in control subjects during the exposure. The ratings increased over time in sMCS subjects and stayed at a low level in control subjects, as in the study by van Thriel et al. (2002).

Female subjects with CI rated ammonia^25^ as more unpleasant and more pungent than female control subjects (Pacharra et al. 2016a; see above).

### Summary of findings

In studies of ‘hours’ exposure, only subjective ratings increased in sMCS subjects compared to healthy controls. Eye blinking frequency as an objective marker of sensory irritation was unaffected, though an exposure-related increase in blinking was seen at the highest exposure level.

In ‘seconds’ exposure, only MCS subjects reported an increase in ratings, but objective markers of sensory irritation remained unchanged. Only one study showed higher eye blinking frequency for exposure to ethyl acrylate (0–40 ppm) than the control condition (Kiesswetter et al. 2005). However, though an objective marker of sensory irritation (blinking frequency) could be related to chemical exposure, there was no moderating effect in sMCS on sensory irritation. It is possible that health risk perception has an influence on perception in MCS patients, as shown in asthmatics to result in moderate perception and airway response according to Jaén and Dalton (2014). Palmquist and Claeson (2022) argue that subjects with building-related symptoms and CI perceive irritation at lower concentrations than others. They hypothesize that the increased sensitivity is due to altered trigeminal reactivity by inflammation or oxidative stress. It cannot be ruled out that this effect is odor-driven. Stress is known to have an impact (lowering) on the (olfactory) detection level of mercaptoethanol (Pacharra et al. 2015). In a recent review, Viziano et al. (2018) conclude that a combination of neural altered processing of sensorial ascending pathways with peculiar personality traits lead to MCS. The results reported here support the influence of personality traits. Thus, the next paragraph deals with the influence of personality traits (affectivity) on the perception of sensory irritation.

## Influence of the grouping factor *affectivity* on sensory irritation

Affectivity is the ability to experience and cope with affects. Such affects can be positive or negative (Watson et al. 1988). For instance, olfactory stimuli can induce mood changes (Seubert et al. 2009), i.e., there is an affective response to odors. Automatic associations about odors indicate implicit attitudes towards odors (Bulsing et al. 2009, 2007). The implicit association test can diagnose such associations (Bulsing et al. 2009, 2007). A few studies about affectivity and sensory irritation have been identified.

### Studies about ‘hours’-exposure

#### Subjective ratings

Lang et al. (2008) used ‘negative affectivity’ (Positive and negative affect scales, PANAS; Watson et al. 1988) as a covariate in their analysis already referred to in the gender paragraph. They argue that at lower formaldehyde concentrations, negative affectivity had a stronger influence than at high formaldehyde concentrations. However, this study suffers from methodological caveats (unbalanced sequence of exposures and unusually high eye blinking frequencies), which hampers the interpretation; however, negative affectivity seems to affect ratings only at low concentrations.

Ihrig et al. (2006) investigated the influence of personal traits on the complaints about ammonia exposure. Subjects were exposed up to 50 ppm ammonia on five subsequent days in an ascending sequence. Besides the PANAS questionnaire, the Freiburger Persönlichkeits Inventar (FPI; Fahrenberg et al. 2010) was used to assess the affectivity. The study showed that positive affectivity was significantly, negatively correlated to the rating of irritative ocular and nasal symptoms (SPES; Iregren 1998) only at low concentrations (10 and 20 ppm). This shows that the higher positive affectivity, the lower the symptom ratings when exposed to low ammonia concentrations. Subscales ‘Nervousness’ and ‘Depression’ from the FPI had a significant positive correlation with ratings of irritation symptoms at low concentrations. The higher the subscale, the higher the ratings of irritation.

Müller et al. (2013) also used the PANAS questionnaire to characterize their subjects. Furthermore, they identified hypo- and hypersensitive subjects with a median split on the rating of a CO_2_-exposure. Subjects were exposed to five different concentrations of formaldehyde on five subsequent days for 4 h. The irritation ratings by the hyposensitive subjects compared to hypersensitive subjects did not differ significantly for the eyes and the nose (SPES). However, results about the affectivity were unreported.

Nordin et al. (2017) exposed subjects to odorous substances (limonene as a pleasant odor and pyridine as an unpleasant odor) for 35 min at levels below their thresholds for sensory irritation but above their odor thresholds. They found that negative affectivity led to higher symptom ratings only when exposed to the unpleasant odor.

Pacharra et al. (2016d) used an implicit association test using odor terms (Bulsing et al. 2007) to identify subjects with negative and positive implicit associations towards odor. Furthermore, they divided the subjects into a low and a high olfactory acuity group (based on Sniffin’ Sticks; Hummel et al. 1997). Both dichotomized implicit association towards odor and dichotomized olfactory acuity, were independent factors that resulted in four experimental groups. The exposure scenario (with ammonia) was the same as in Pacharra et al. (2016b; see above). Subjects with strong positive associations towards odor rated ammonia as more pungent than those with weak positive automatic associations towards odor only in the subgroup with low olfactory acuity.

### Summary of findings

Both MCS and (negative) affectivity result in higher ratings of irritation at low concentrations. At higher concentrations, the ratings of MCS and negative affectivity subjects and control subjects converge. The exposure concentrations are generally far below the threshold for sensory irritation in most of the studies. In one study differences between MCS and control subjects could not be observed in the eye blinking frequency at the highest exposure (Kiesswetter et al. 2005). Thus, odor perception appears to play a central, but not exclusive role, in reporting irritation.

It is well known that expectations, beliefs, information, and biases have an impact on odor perception and reported irritation symptoms (Dalton and Jaén, 2010; Jaén and Dalton 2014), as previously suggested by Das-Munshi et al. (2006). Several psychological characteristics are found in idiophatic environmental intolerance patients (Papo et al. 2006).

Bornschein et al. (2002) did not observe any deviation from norms between 12 MCS-patients and 12 matched controls in a positron emission tomography study where cerebral glucose metabolism was an indicator of cerebral dysfunction. Chemical intolerance seems to be associated with disease comorbidities (Palmer et al. 2021). However, a causal relationship has not been identified.

The influence of psychological and physiological components on the report of sensory irritation is difficult to assess (Rossi and Pitidis 2017; Zucco and Doty 2022). The influence of expectations and beliefs, however, has not been found in objective markers of sensory irritation. Nevertheless, the perception of strong symptoms even at low concentrations of volatile substances should be considered in risk assessment. Thus, the complex (top-down) psychological effects of odorous (pleasant or unpleasant) substances should be assessed, if complete or partially-complete protection of the general population should be the target. This, however, is challenging since odor thresholds are orders of magnitude lower than thresholds for sensory irritation (Cometto-Muñiz and Abraham 2016). In this case, perceptual ratings, still, are the only possibility to quantify differences in sensitivity for MCS or negative affectivity individuals.

## General discussion

In the regulation of volatile substances, sensory irritation is an important endpoint (Brüning et al. 2014; Nielsen and Wolkoff 2017).

There are at least three obvious factors that have an impact on sensory irritation elicited by volatile substances:

1. volatile substance at a certain concentration
2. situational context/demands
3. individual differences

Different volatile substances have different water solubilities (cf., Garcia et al. 2009; Shusterman 2002) and, therefore, different deposition rates along the airways that determine the target site at which sensory irritation takes place. At target sites, different channels (i.e., TRPA, TRPV, GPCR, ASIC; Lehmann et al. 2017; Shusterman 2007) might lead to sensory irritation. High concentrations of substances, however, may reach ‘lower’ target sites depending on the deposition capacity. Furthermore, for lipophilic substances, the protective perceived irritation in the upper airways may be absent. Thus, they can cause injury at high concentrations. Risk assessment should handle such lipophilic substances with special care in a substance-by substance approach.

However, the target site of a volatile substance also depends on situational influences like mode of breathing, i.e., nasal breathing versus oral breathing, e.g., due to the physical workload. Physical workload might lead to more oral breathing resulting in a higher fraction of volatile substances reaching ‘lower’ target sites. Individual variance might be higher or lower for one channel and vice versa.

This manuscript (1) reviewed empirical evidence about differences between groups in the general population and (2) aimed at deriving an AF for the general population. Wherever possible, controlled human exposure studies have been considered. The results of the reviewing process are summarized in Table 6.

**Table 6:** Summary of reviewed empirical human exposure studies about group differences regarding sensory irritation

Grouping factor	Empirical bases	Decidedness of human exposure studies		Concerns	Proposed AF
		Objective	Subjective		
Gender	29 studies	3/24 studies show higher sensitivity in females (hours and seconds exposures)	4/22 studies show higher sensitivity in females (hours and seconds exposures)	Differences only at the nose	Sensitivity of females might be higher
			Hour’s exposure: sensory irritation is uncertain		Substance-specific AF 2 for gender (age, lifestyle and disease), also proposed by Nielsen and Wolkoff (2017)
*Age*					
Older people	10 studies	4/7 studies show lower sensitivity in older people (seconds exposure)	1/4 studies show lower sensitivity in older people (hours and seconds exposure)	Warning signals and clearance might be impaired	Sensitivity of older people is lower
				Detrimental effects of lower concentrations at longer exposures are possible	It remains unclear whether an AF is needed due to possible detrimental effects in the long run
					No AF can be proposed
Children	1 study	No higher sensitivity of children aged 7 and older		Children up to 5 years were not studied	Experimental studies are difficult
		Unclear whether children aged 5–7 years did understand the task		Only one substance	Models incorporating developmental processes are needed to assess altered sensitivity in children
				Developmental changes in involved systems are likely	No AF can be proposed
*Diseases*					
Asthma/allergies	8 studies	1/4 studies show higher sensitivity in asthma / allergy (seconds and minutes exposure)	1/4 studies show higher sensitivity in asthma / allergy (seconds, minutes, and hours exposure)	Self-selection of participants	Undoubtedly, certain diseases impair sensory processes
			Allergic subjects were studied outside the pollen season		Kinetic and dynamic modelling of sensory irritation in diseases is necessary
			Mucus production might attenuate sensory irritation, but also trap water soluable substances		For asthmatics large differences in sensitivity could be demonstrated for single substances (three- to nine-fold), however, confounded
COPD			Sensory irritants might cause or aggravate asthma/allergies even at low concentrations		AFs for different substances (substance classes) seem more supportable than a general AF of 10 or more
Others					No AF can be proposed
*Vulnerability*					
MCS/CI	12 studies	0/5 studies show higher sensitivity in MCS/CI (hours, minutes, and seconds exposure)	8/10 studies show higher sensitivity in MCS/CI (hours and seconds exposure)	Most of the differences found refer to low substance concentrations	MCS/CI and affectivity fail in predicting objective differences in sensitivity
				Ratings converge at higher concentrations	Effects were seen foremost at low concentrations (without sensory irritation)
				No differences in objective measures could be demonstrated	A physiological specification for MCS/affectivity has not been identified, yet
					Subjective suffering might be high
					No AF can be proposed
Affectivity	5 studies		3/5 studies show higher sensitivity in negative affectivity	Studies focus on subjective ratings of irritation	
			1/5 studies show higher sensitivity in positive affectivity	Differences in subjective ratings foremost at low concentrations	

### Evaluation of differences identified by grouping factors

The first aim of this study is to identify relevant (human exposure) studies that could be a platform for derivation of (an) AF(s), which is specific for sensory irritation in the general population.

Individual differences in sensory irritation depend on the sensory receptors, the transduction, and the strength/evaluation of the perception.

For instance, the density of receptors differs across lifespan (with a maximum in healthy adolescents and at healthy young adulthood). Further, some diseases might influence sensory density (i.e., chronic cough; Shapiro et al. 2021). However, there are the body´s own defense mechanisms (i.e., eye blinking, nasal mucociliary clearance). These also depend on individual differences that might differ over the lifespan and may be hampered by diseases; thus, they may influence sensory irritation.

A first step to capture individual differences in the general population is to take a closer look at sub-groups known to represent such differences, i.e., gender, age groups, patients suffering from diseases, and subjects that consider themselves as sensitive. This first step is carried out in this study.

#### Gender

The grouping factor *gender* has the best empirical base of all reviewed grouping factors. The influence of gender on sensory irritation has been investigated on a variety of endpoints at different time scales. Females report more symptoms in the eyes in a few studies. However, no gender differences could be found in objective markers of sensory irritation in the eye. Females seem to be more sensitive than males when exposing the nose for seconds in some studies, but an odor-mediated bias cannot be excluded in every significant study. Therefore, it cannot be ruled out that methodological shortcomings have an impact on the differences. Many studies did not find convincing evidence for gender differences at all. The reviewed studies are unable to disprove an AF of 2 as proposed by Nielsen et al. (2007; Nielsen and Wolkoff 2017) because it must incorporate not only the influence of gender, but also the additional influences of age, lifestyle, and diseases, and in some cases climatic conditions. The reviewed studies are representative of first and foremost young, healthy, non-smoking subjects, and the investigated substances. Therefore, substance-specific AFs (for gender) might be more adequate than a general AF for gender influence.

#### Age

The influence of age on sensory irritation is ambiguous. Obviously, is it reasonable to distinguish between older people and young children in a risk assessment.

##### Older people

Based on the reviewed studies (n = 10), older people are less sensitive than young adults. However, it seems that high age is not necessarily a causal influence but could be a correlative effect on sensory (or olfactory) loss in older people. As there is a high variability as well in the young adult groups as in the older groups, age-related pathologies may be responsible for the effect of older age (Stevens et al. 1982). However, older people as a group are less sensitive to sensory irritation. An AF for older age, therefore, seems unnecessary at first glance. Anyway, caution is advised. Lesser sensitivity might result in slower, weaker, or missing physiological defense processes against sensory irritants, i.e., less eye blinking (Sharma and Hindman 2014; Wolkoff 2020, 2017), or lower epithelial beat frequency (Ho et al. 2001). Especially, detrimental effects arising from weaker own-body protection might come up after longer exposures of hours or days (which are rarely/not captured in the reviewed studies). Objective age effects are only seen in threshold studies (seconds exposure) with higher thresholds in older people. Only one study investigated exposures longer than seconds (4 h exposure of 20 ppm 2-ethylhexanol; Schäper et al. 2015). No age effect was observed in eye blinking, though 2-ethylhexanol triggered more blinking during the highest exposure condition (20 ppm) in both young and older subjects (45–67 years). However, it remains unclear whether there could be age effects at longer exposure times, older ages, with other substances or deteriorating climatic conditions. A temporal summation (over longer periods) due to insufficient defense mechanisms in older people cannot be excluded. One long-standing question is whether a less stable eye tear film, as encountered in older people (especially women), is more vulnerable to sensory irritation, i.e., the no-observed-adverse-effect-level for a given irritant would be lower than in younger people with a healthy and stable tear film. Therefore, an empirically based quantification of a risk factor for older people is not possible.

##### Children

Experimental studies investigating the sensitivity of young children are scarce (n = 1). At the age of 7 years, children show no significantly lower sensitivity than older subjects when lateralizing eucalyptol in the nose. It is not clear whether younger children have a higher sensitivity. As experimental studies are difficult from an ethical perspective, other approaches are necessary to derive AFs for young children. For sensory airway irritation, Frank et al. (2018) identified so-called KEs and MIEs, which describe initial points of interaction with a volatile substance that result in a perturbation / sensory irritation. Several studies have shown developmental changes in processes involved in MIEs.

Therefore, KEs leading to sensory irritation by volatile substances binding to the TRPA1 receptor might be moderated by developmental processes at a young age. Thus, developmental processes might influence the perception of sensory irritation at a given concentration. To make it more complicated, the organism reacts to defend itself against harmful volatile substances; for instance, eye blinking to maintain the tear film stability and to clear the ocular surface and mucus production to remove harmful substances from the airways (mucociliary clearance). Such defense mechanisms allow for coping with volatile substances that could evoke sensory irritation. However, if these mechanisms are impaired, volatile substances could lead to sensory irritation at concentrations that otherwise would be tolerable for young healthy adults. Usually, there is more than one cooperating defense mechanism. For example, infants show lower eye blinking frequency (less than one time a minute). On the other hand, their eye tear film is more stable than in adults. Thus, there is a complex interplay of defense mechanisms involved in coping with sensory irritants. A comprehensive modelling of such mechanisms (i.e., PD/PK models) regarding developmental changes would be helpful for risk assessment as it allows for indirect assessment of exposure effects via ‘critical mass’ at the trigeminal system.

Based on what is known about developmental processes up to now, an AF could not be derived.

#### Health status

Target sites for volatile irritants are also impaired in several diseases (i.e., allergic rhinitis, asthma, COPD). Diseases might also influence the kinetics and dynamics of the sensory irritant. However, empirical evidence by experimental exposure is limited to diseases. Most likely, patients with severe diseases refrain from participating in exposure studies. Consequently, empirical evidence is limited to patients with mild to moderate course of disease and cannot be generalized. Furthermore, many diseases might have a direct (higher sensitivity at target sites) or indirect impact (hampering or intensifying defense mechanisms) on sensory irritation. The impact of diseases on sensory irritation has only been investigated in a few studies. Again, comprehensive models of impaired target sites and suspended defense mechanisms would be helpful. In the case of allergy, allergen-induced neuromodulations are accurately described (cf., Undem and Taylor-Clark 2014). Furthermore, AOPs of allergy development already exist (i.e., in the case of chemical respiratory allergy/occupational asthma; Kimber et al. 2014). What is missing are models that allow for a quantification of, for example, differences between healthy and asthmatic people in sensory irritation. Ideally, the severity of the disease (i.e., via RAST classes in allergy) should be considered in such models that might be expanded to comorbidities. It would be desirable that such models consider different degrees of impairment at target sites and defense mechanisms (dynamic components), which depend on the diseases. The magnitude of impairment can probably be mapped to the severity of disease. Prevalence of diseases should be considered for the derivation of an AF for the whole population, if possible, even regarding the severity (i.e., RAST classes in allergy). It is worth mentioning that long-term exposure to volatile substances at concentrations not generating sensory irritation might have an adverse impact (exacerbation of asthma / increased probability to develop asthma) in susceptible groups (asthmatics / children) of the general population. In risk assessment, such a potential hazard should be considered for each substance. Again, QSAR models of respiratory sensitizers (i.e., Graham et al. 1997) would be helpful.

On the other hand, the disease asthma appears to exhibit an indirect protection against water-soluble irritants due to the excess mucus production in the nasal cavity and throat (cf., Fadeyi et al. 2015; Golden 2011; Johansson et al. 2017; Larsen et al. 2013; Wolkoff and Nielsen 2010).

Sensory irritants may cause or aggravate asthma/allergy symptoms (Baur et al. 2012; Briatico-Vangosa et al. 1994; Nurmatov et al. 2015). Thus, risk assessment for sensory irritation can be more complicated, even at concentrations far below the irritation threshold among subjects suffering from low affectivity or allergies. Odor-mediated reactions cannot be ruled out, cf., Cometto-Muniz and Cain (1991).

#### Vulnerability

There is a large individual variety in the perception of volatile substances. The same volatile substance that causes an irritant perception in one person might be barely noticed by another person (Dalton and Jaén, 2010). This difference can be clearly observed in MCS patients (n = 12 studies reviewed). The exposure concentrations are far below sensory irritation but above the threshold for odor perception in most studies about MCS. MCS subjects show higher perceptual and symptom ratings compared to control subjects, but MCS patients often perceive the odor more intensely, while the odor thresholds among MCS patients and healthy people do not differ (We referred to such an effect as the third manifestation of sensitivity in *‘*Manifestations of sensitivity’).

Why some odors evoke health symptoms is not completely understood but some processes might be responsible (Schiffman and Williams 2005), like affectivity, modulation of breathing, exacerbation of diseases by inducing stress and impairing mood, and learned associations (Jaén and Dalton 2014). Further factors influencing odor perception were summarized by Wolkoff (2013; cf., Table 3).

It is not possible nor reasonable to develop an AF that accommodates a sensitivity triggered, solely, by odor perception, since odor thresholds generally are orders of magnitude lower than thresholds for sensory irritation (Cometto-Muñiz and Abraham 2016).

Adequate risk communication might mitigate such perceptual differences (Dalton and Jaén, 2010; Jaén and Dalton 2014; Nordin 2020).

### AF for the general population

In addition to finding differences in sensitivity to sensory irritation between groups of the general population, the second aim of this study was the derivation of an AF for the general population considering vulnerability and susceptibility factors. This aim could not be achieved due to several reasons:

1. Lack of an empirical base (children, health status)
2. Lack of representativity / generalization (health status, children, vulnerability)

- Self-selection of subjects (health status, MCS, affectivity)

(3) Time aspects (older people)

- Influence of altered warning signals / clearance (older people, children)
- Potential of volatile substances to elicit or aggravate diseases (e.g., asthma, allergies)

(4) Absence of objective indicators of sensory irritation in many studies (most notably, vulnerability) or trigger olfactory effects

The investigated groups, however, might differ by other variables than the grouping factors. This may influence the outcome according to the study by Rosenkranz et al. (2020), who demonstrated that a fraction of otherwise ‘healthy’ subjects showed signs of diseases. Thus, study results may be influenced by unrecognized and undiscovered illnesses. Furthermore, psychological effects, inter alia expectations or stress might affect airway physiology (Jaén and Dalton 2014; Pacharra et al. 2015). In addition, methodological shortcomings may underestimate the odor potencies of a substance as they increase the variability and therefore inflate what is assessed as individual differences (Cain and Schmidt 2009).

#### How to address the identified problems in risk assessment

##### Variety of target sites

In animal studies, extrapolation factors were used to extrapolate from subacute to subchronic exposures and from subchronic to chronic exposures. For example, a multitude of 226 different extrapolation factors have been identified for 71 volatile substances by extrapolation from subchronic to chronic effects in inhalation (Escher et al. 2020). The variance in these factors shows confounders like the uncertainty of identified no-observed-adverse-effect-levels and differences in study design.

It seems reasonable to establish AFs that are target site specific. If a volatile substance at a certain concentration only affects the eye, gender variance at the nose does not necessarily have to be considered. The same might be true for other target sites.

Therefore, QSARs (Abraham et al. 2016, 2007, 1998, 1996; Alarie et al. 1996; Cometto-Muñiz et al. 1998; Cometto-Muñiz and Abraham 2016; Cronin et al. 2003; Hau et al. 1999) could be a tool to refine grouping of substances (European Chemicals Agency 2021). The consequence of such grouping procedures could be a variety of AFs for substance groups. The derivation of an AF would require knowledge about the concentration/mass of sensory irritant at a specific target site (kinetic processes); and in the end, the defined target of percentage protection of the population.

##### Individual /intraspecies factors

In animal studies, intraspecies factors are usually not considered at all. Risk assessment for the general population, however, must consider susceptible and vulnerable groups with individual factors, i.e., children or persons suffering from chronic diseases.

This review could propose an AF only for the grouping factor gender. Other grouping factors describe other sensitivities in sensory irritation. Interaction between such grouping factors are also possible. Johansson et al. (2016), for example, identified a ninefold higher sensitivity of asthmatics to sulfur dioxide. However, a general AF of 10, as proposed by the authors, could be valid only for pulmonary irritants (like sulfur dioxide). A substance-specificity in AFs, therefore, is more reasonable from an economic point of view than a general AF. Models must be developed that incorporate differences in susceptible and non-susceptible people exposed to the same concentration of the same volatile substance (QSAR group). It must be emphasized, however, that justifying and deriving AFs for susceptible people would require a detailed analysis of causation at the individual level, cf., Haanes et al. (2020). Furthermore, it must be considered that the efficiency of defense mechanisms (dynamic processes) also could be differential.

##### Temporal aspects of exposure

The duration of the volatile substance exposure is relevant. Controlled human exposure studies use different experimental situations to investigate individual differences in human sensory irritation, ranging from seconds- to hours-exposure and – rarely—to exposure on subsequent days. However, sensory irritation underlies temporal summation (effects of up to tens of minutes have been observed; Cain et al. 2010). Therefore, it is reasonable to assume that hours-exposures should reveal any sensory irritation effect. In human exposure studies over hours, only a few concentrations can be tested. Due to ethical reasons, the highest concentration is not allowed to exceed legal OEL values. Therefore, harmless concentrations might even be higher (cf., Mangelsdorf et al. 2020) and individual differences would not come into effect. Therefore, seconds- and minutes-exposures are relevant, too.

In risk assessment, exposure effects found in seconds- to hours-exposures must be extrapolated to, in the worst case, lifelong exposure. It is unclear whether defense mechanisms would work properly if they were challenged continuously over the years. If not, it could be speculated that volatile substance concentrations repeatedly over hours might induce sensory irritation after longer-lasting exposures without sufficient exposure-free periods for recovery. Such a situation, however, appears only hypothetical for a major part of the population.

The relationship between defense mechanisms / clearance in response to continuous volatile substance exposure should be further investigated.

The potential of volatile substances to elicit allergies/asthma at low concentrations must be considered in risk assessment. Reliable models of such pathogenesis are necessary.

##### Situational contexts of exposure

Changing from nasal to oral breathing (dynamic component) leads to a circumvention of the nose and its scrubbing (kinetic) effect. Therefore, there might be special AFs, i.e., for sports halls and playgrounds / schoolyards (regarding diseases and maybe also for hospitals / retirement homes). Furthermore, visual demands might lead to higher vulnerability of dry eyes because of less or incomplete eye blinking (Wolkoff 2020).

Coping with different situational contexts depends on individual prerequisites. Therefore, when considering individual differences in sensory irritation and proposing AFs, substance and situational factors must be assessed, too (cf., Haanes et al. 2020).

### Model requirements for risk assessment

Comprehensive modeling with kinetic (delivery to target site) and dynamic (action and reaction at target site) components (i.e., PBPK^26^, PD/PK^27^, TD/TK^28^) could be used for subgroup-specific risk assessment (i.e., Poet et al. 2010). While C^n^ x t models require a non-interrupted exposure duration (Pauluhn 2019) with a stable influence of dynamic and kinetic processes, realistic exposure can be intermittent. Thus, in some phases, one of the processes will prevail (leading to the summation of substance or clearance). Comprehensive modelling (i.e., PD/PK models) of the concentrations at target sites is required in evidence-based risk assessment for sensory irritation. Such models should consider the kinetic component (delivery to target side) as well as the dynamic component (action at target site and physiological responses like defense mechanisms) in sensory irritation as well as the effects of continuous exposure. For systemic effects, gender and age differences in biochemical processes have been considered, e.g., in PB/PK models of 2-butoxyethanol disposition in rats and mice (Corley et al. 2005).

Similar models for sensory irritation are still lacking. A ‘critical’ mass of substances at the specific receptors that leads to sensory irritation should be identified (trigeminal system as a mass detector rather than a concentration detector; Frasnelli et al. 2017; Hummel and Frasnelli 2019; Kleinbeck et al. 2020). Deliverance to the receptor as well as clearance at the receptors might be influenced by age effects at longer exposure and as a result may influence the critical mass at the receptor. One must keep in mind that not only the receptors but also nerve transmission might be affected (nerve damage in chronic sinusitis, Auais et al. 2003; i.e., developmental changes in children, Uppal et al. 2016). Nevertheless, exposure studies with potentially susceptible subjects are also needed to evaluate the adequacy of the PD/PK modeling and to relate differences in the response of the target site to differences in objective and subjective markers in sensory irritation in vivo.

## Conclusion

Though recent publications propose AFs for asthmatics and MCS patients, respectively, (10; Johansson et al. 2016) and the general population (20; Mangelsdorf et al. 2020), this review was unable to identify supporting and meaningful empirical evidence for an AF greater than 2 for protection of the susceptible subgroups of the general population, solely based on human exposure studies. This implies it can be for some substance between 1 and 2. Asthmatics and healthy controls were differently affected only by a few substances in the review by Johansson et al. (2016). However, Johansson et al. considered substances that were either pulmonary irritants or both sensory and pulmonary irritants (e.g., sulfur dioxide), while Mangelsdorf et al. (2020) applied a general precautionary AF of 20 without scientific support. Thus, it is recommended to apply an evidence-based risk assessment approach to be carried out, substance by substance. QSARs can be used to classify substance classes for substance- and pathway-specific AFs (Falk-Filipsson et al. 2007). Furthermore, contexts of exposure must be considered. Other AFs might apply for workplaces where dry air, high room temperature, and visually demanding tasks (e.g., video display unit work) aggravate the eye tear film stability, rather than for places in which workload is balanced. Indoor playgrounds might need other AFs due to physical workload and affected groups of the general population.

Extra emphasis should be put on populations such as neonates and genetically sensitive subgroups, fetuses, and children, because they may be particularly susceptible or vulnerable during development and maturation. Gender differences in sensitivity, deficiencies in the databases, nature of the effect, duration of exposure, and route-to-route extrapolation need also to be considered. Since AFs are used to compensate for lack of knowledge, we consider that it is prudent to adopt a conservative approach. When new empirical knowledge appears that reduces the uncertainties, this should be incorporated into a new risk assessment leading to more evidence-based AFs. While gender differences could be considered by a substance-specific conservative AF of 1–2 (incorporating also influences of age, lifestyle, and diseases, as proposed by Nielsen and Wolkoff 2017), empirical and supporting evidence for the development of other AFs (for children, older persons, patients suffering from diseases) is lacking.

Though older people as a group have higher thresholds for sensory irritation, other factors, like defense mechanisms might be impaired by diseases that could induce more susceptibility to long-term effects of volatile substance exposure.

For children, there might be time slots in their development, in which they might be more susceptible or vulnerable to sensory irritation than adults. However, children might even be less susceptible (cf., higher tear film stability) than adults. Most likely, there is still development in sensory systems from birth to the age of 7 years that might be covered by an AF. However, a quantification based on empirical data is not possible. PD/PK models representing developmental changes could be established for an ‘empirical’ basis of an AF.

For patients suffering from diseases the derivation of an AF from empirical knowledge is also difficult. Many diseases have an impact on sensory systems resulting in sensitization (that might depend on the severity of the disease). On the other hand, increased mucus production due to diseases could even protect the airways from sensory irritation, but such a protective effect of sensory irritation, probably, is an exception for highly water-soluble substances; exaggeration of sensory irritation by disease is more common. Volatile substances might even elicit diseases like allergies/asthma. Again, comprehensive PD/PK models of patients with diseases (representing disease severity) considering the impact of volatile substances in pathogenesis would be desirable to achieve an ‘empirically-justified’ AF.

There is also a lack of knowledge about the impact of affectivity/MCS on sensory irritation (and the inevitable associated olfactory impact). At first glance, both seem to influence the perception of low concentrations of volatile substances. One must keep in mind that the exposure concentrations were far below sensory irritation thresholds in most of the studies. At higher concentrations (still below the sensory irritation threshold) such differences compared to’normal’ subjects decrease. Based on the reviewed studies, a special AF for sensory irritation regarding affectivity/MCS is not justified. Nevertheless, the influence of affectivity/MCS on olfactory loads might be considered in setting exposure limits, however challenging, if possible beyond the individual level. Thus, for OELs perceptual (olfactory) effects of a substance are not health effects. Adverse exposure effects at workplaces require ‘(a) sensory irritation, (b) considerable odor annoyance or (c) in individual cases’odor associated’ symptoms must occur’ (List of MAK and BAT Values 2021, https://series.publisso.de/sites/default/files/documents/series/mak/lmbv/Vol2021/Iss2/Doc002/mbwl_2021_eng.pdf). Therefore, if the personality factor affectivity/MCS elicits ‘considerable odor annoyance’ or ‘odor-associated symptoms’ at lower concentrations than in groups with different personality, this must be dealt with case-by-case at the individual level. However, up to now, MCS is far from being a well-defined symptom class caused by known special properties of volatile substances at specific concentrations. Protection of MCS patients by evidence-based AFs is not possible, and other mitigation strategies should be considered.

It is reasonable to use substance-specific AFs, since differences in sensitivity might be limited to one sensory system and the qualititative and quantitative database for sensory irritation might be different. For example, highly water-soluble substances are scrubbed by the nose and do not reach the middle or lower airways. Further, situational factors might lead to a circumvention of a sensory system (e.g., mouth breathing in a high physical workload). Therefore, it is reasonable to consider situational factors in substance-specific AFs; furthermore, the influence of climatic conditions may be considered.

Comprehensive models of sensory irritation comprising developmental differences, changes caused by diseases, and age-related decline could bridge the gap of knowledge caused by ethical concerns to expose children to volatile substances, experimentally, and by self-selection in experimental studies with patients suffering from diseases. Physiochemical properties like water solubility and QSARs could help to categorize substances eliciting sensory irritation with respect to a target site (e.g., eyes, nose, middle, and lower airways) and mode of action at the target site.

## Characteristics of the reviewed studies

See below Table 7.

**Table 7:** Characteristics of the reviewed studies

No	Study	Substance	CAS	Water solubility^a^	Substance delivery	Subjects		Age	Max. concentration	Comparison	Measures of sensory irritation
						Female	Male				
[1]	Ernstgård et al. (2002)	2-propanol	67–63-0	Miscible	Exposure lab	28	28	20–49	150 ppm	Clean air	VAS^b^, eye blinking frequency, spirometry, rhinometry, NALF^c^, color vision
		m-xylene	108–38-3	0.174 g/l (25° C)					50 ppm		
[2]	Gminski et al. (2011)	Fresh OSB^d^ Emissions	–	–	Exposure lab	11	13	21–31	8.9 mg/m3^e^	Storage for 2 and 8 weeks	Spirometry, exhaled NO, eye blink frequency, VAS
[3]	Hey et al (2009)	Propionic acid	79–09-4	370 g/l (20° C)	Exposure lab	12	11	25.7 (SD 3.9)	10 ppm	0.3 ppm, 5 ppm	LMS^f^, SPES^g^, neurobehavioral tests
[4]	Juran et al. (2012)	Cyclohexylamine	108–91-8	Miscible	Exposure lab	12	12	20–36	10 ppm	1 ppm, 0–4 ppm	LMS, SPES, eye blinking frequency, neurobehavioral tests
[5]	Kleinbeck et al. (2008)	Ethyl acetate	141–78-6	85.3 g/l (20° C)	Exposure lab	24	n.a		0–800 ppm	2 ppm, 400 ppm	LMS, SPES, eye blinking frequency, rhinomanometry, neurobehavioral tests
[6]	Kleinbeck et al. (2017)	Ethyl acrylate	140–88-5	20 g/l (25° C)	Exposure lab	10	9	19–32	0–10 ppm	0 ppm, 2.5 ppm, 0–5 ppm, 5 ppm	LMS, eye blinking frequency, rhinomanometry, NALF, neurobehavioral tests
[7]	Pacharra et al. (2017)	Ammonia	7664–41-7	531 g/l (20° C)	Exposure lab	19	18	23.6 (SD 2.5) and 25.1 (SD 3.9)	0–40 ppm	2.5 ppm	LMS, SPES, eye blinking frequency, rhinomanometry, exhaled NO, NALF
[8]	Pacharra et al (2016c)	Propionic acid	79–09-4	370 g/l (20° C)	Exposure lab	22	26	26.4 (SD 0.9) and 24.6 (SD 0.7)	0–20 ppm	0.3 ppm	LMS, SPES, eye blinking frequency, neurobehavioral tests
[9]	Schäper et al. (2015)	2-ethylhexanol	104–76-7	0.6 g/l (20° C)	Exposure lab	17	15	18–67	20 ppm	1.5 ppm	LMS, SPES, rhinomanometry, eye blinking frequency, NALF, neurobehavioral tests
[10]	Sucker et al. (2019)	Ethyl acrylate	140–88-5	20 g/l (25° C)	Exposure lab	11	11	25	0–10 ppm	0.05 ppm, 5 ppm	LMS, eye blinking frequency
[11]	van Thriel et al (2010)	Sulfur dioxide	7446–09-5	112.7 g/l (20° C)	Exposure lab	8	8	24.3 (SD 5.2) and 28.4 (SD 3.9)	2 ppm	0 ppm, 0.5 ppm, 1 ppm	LMS, SPES, rhinomanometry, eye blinking frequency, spirometry
[12]	Wålinder et al. (2008)	1-octen-3-ol	3391–86-4	1.31 g/l (20° C)	Exposure lab	14	15	20–54	10 mg/m3	0 mg/m3	VAS, eye blinking frequency, BUT^h^, vital staining (eye), rhinometry, NALF, transfer test, spirometry
[13]	Sundblad et al. (2004)	Ammonia	7664–41-7	531 g/l (20° C)	Exposure lab	7	5	21–28	25 ppm	5 ppm	VAS, spirometry, exhaled NO, NALF, blood cell counts
[14]	Shusterman et al. (2005)	Acetic acid	64–19-7	miscible	Nasal CPAP Mask	8	8	21–65	15 ppm	0 ppm	VAS, rhinomanometry
[15]	Yang et al (2001)	Formaldehyde	50–00-0	400 g/l (20° C)	Small chamber	4	4	21.9 (SD 5.9)	4.4 ppm	1.7 ppm, 3.0 ppm	VAS, eye blinking frequency
[16]	Claeson and Nordin (2011)	Amyl acetate	628–63-7	1.7 g/l (20° C)	Dynamic olfactometer	12	12	20–35	3.8 ppm	Ascending from 1.6 ppm	Threshold test, Borg scale
[17]	Dalton et al. (2000)	Methyl isobutyl ketone	78–93-3	292 g/l (20° C)	Glass bottles	22	18	18–65	6.25% v/v	18 dilutions	Lateralization threshold, LMS
[18]	Frasnelli and Hummel (2003)	Menthol	89–78-1	456 mg/l (25° C)	Olfactometer	8	8	18–35	n.a	12 concentrations	NMP^i^, lateralization thresholds
		Linalool	78–70-6	1.59 g/l (25° C)					n.a	12 concentrations	
[19]	Hummel et al. (2003)	Benzaldehyde	100–52-7	6.95 g/l (25° C)	Squeeze bottles	8^j^	16	19–24	n.a	40 stimuli	Lateralization threshold
		Eucalyptol	470–82-6	3.5 g/l (21° C)							
[20]	Kleinbeck et al. 2011	Sulfur dioxide	7446–09-5	112.7 g/l (20° C)	Flow-olfactometer	22	22	20–44	33.6 mg/m3	Ascending from 0.2 mg/m3	LMS, breathing depth
[21]	Mattes and DiMeglio (2001)	Ethanol	64–17-5	Miscible	Sample bottles	25	25	21–50	7.5 × 10–3% v/v	Starting from 1.0 × 10–6% v/v	Lateralization threshold
[22]	Stuck et al. (2006)	Eucalyptol	470-82-6	3.5 g/l (21° C)	Dynamic olfactometry	42	53	18–80	n.a	40 stimuli	Lateralization threshold, ERP^k^
[23]	van Thriel et al. (2006)	Formic acid	64–18-6	Miscible	Glass bottles	144^l^	144^l^	26.6 (SD 5.0), 28.0 (SD 4.5), 24.1 (SD 4.7), 25.1 (SD 5.8)^l^	n.a	20 stimuli	Lateralization threshold
		Acetic acid	64–19-7	Miscible							
		Propionic acid	79–09-4	370 g/l (20° C)							
		Cyclohexylamine	108–91-8	Miscible							
		Dimethylamine	124–40-3	Miscible							
		Trimethylamine	75–50-3	Miscible							
		Ethyl formate	109–94-4	105 g/l (20° C)							
		Ethyl acetate	141–78-6	85.3 g/l (20° C)							
		Ethyl acrylate	140–88-5	20 g/l (25° C)							
		Methylcyclohexanone	583–60-8	15 g/l (20° C)							
		Cyclohexanone	108–94-1	103 g/l (25° C)							
		Cyclohexanole	108–93-0	40 g/l (20° C)							
		Ammonia	7664–41-7	531 g/l (20° C)							
		Hydrochloric acid	7647–01-0	720 g/l (20° C)							
[24]	Wise et al. (2007)	n-ethanol	64–17-5	Miscible	Air dilution olfactometer	16	10	22–55	4500 ppm	1800, 2250, 2800, 3550 ppm	Lateralization threshold
		n-butanol	71–36-3	77 g/l (20° C)					1645 ppm	470, 635, 870, 1200 ppm	
		n-hexanol	111–27-3	5.9 g/l (20° C)					16.3 ppm	2.5, 3.6, 5.3, 7.7, 11.2 ppm	
[25]	Ohla and Lundström (2013)	Menthol	89–78-1	4.56 g/l (25° C)	Glass bottle	15	14	20–32	n.a		Lateralization threshold, ERP
[26]	Olofsson and Nordin (2004)	Pyridine	110–86-1	Miscible	Vapor dilutions by flow	19	17	27.2 (SD 3.1) and 25.6 (SD 1.5)	19% v/v	13% v/v, 15% v/v	ERP, Borg scale
[27]	van Thriel et al. (2008)	Acetic acid	64–19-7	Miscible	Flow olfactometer	20	19	18–67	23.8 ppm	9 stimuli	LMS
		Propionic acid	79–09-4	370 g/l (20° C)					19 ppm		
		Formic acid	64–18-6	Miscible					38.2 ppm		
		Ethyl acetate	141–78-6	85.3 g/l (20° C)					580 ppm		
		Ethyl formate	109–94-4	105 g/l (20° C)					7200 ppm		
		Cyclohexylamine	108–91-8	Miscible					260 ppm		
[28]	Gui et al (2014)	Thermal pain				26	27	28.9 (SD 5.3) and 28.3 (SD 7.2)			
[29]	Kleinbeck et al. (2020)	Ethyl acrylate	140–88-5	20 g/l (25° C)	Exposure lab	16	14	19–35	0–10 ppm	0 ppm	LMS, SPES, eye blinking frequency, rhinomanometry, NALF, neurobehavioral tests
[30]	Lang et al. (2008)	Formaldehyde	50–00-0	400 g/l (20° C)	Exposure lab	11	10	18–40	0–1 ppm	0.15 ppm, 0.3 ppm, 0–0.6 ppm	Conjunctival redness, rhinomanometry, spirometry,Vienna test system, SPES
[31]	Wysocki et al. (2003)	1-butanol	71–36-3	77 g/l (20° C)	Glass bottle	142		20–89	n.a.^m^	26 stimuli	Lateralization threshold
[32]	Fadeyi et al (2015)	Ozone (and its initiated limonene reaction products)	10028–15-6	0.57 g/l (20° C)	Exposure Lab	24	14	18–26	20–37 ppb^n^	n.a	Ratings, simulated office tests
[33]	Shusterman et al. (2003b)	Chlorine	7782–50-5	7.3 g/l (20° C)	Nasal CPAP Mask	28	24	18–69	1 ppm	0 ppm	VAS, rhinomanometry
[34]	Shusterman et al. (1998)	Chlorine	7782–50-5	7.3 g/l (20° C)	Exposure lab	8	8	18–40	0.5 ppm	0 ppm	VAS, rhinomanometry
[35]	Khosravi et al. (2014)	Hot air				13					
[36]	Petrova et al. (2008)	Ammonia	7664–41-7	531 g/l (20° C)	Nasal cannula, goggles	40	29.7 (SD 10.8) and 29.1 (SD 9.6)	500 ppm	20 steps from 2 ppm	Lateralization threshold	
[37]	Koskela et al. (2000)	Mannitol powder				17	13	34 (SD 14), 34 (SD 14) and 28 (SD 6)			
[38]	Kiesswetter et al. (2005) / van Thriel et al. (2005)	2-ethylhexanol	104–76-7	0.6 g/l (20° C)	Exposure lab	24^o^	24 (SD 3.7)	0–40 ppm	0 ppm, 0–20 ppm	SPES, Eye blink frequency	
[39]	van Thriel et al. (2002)	2-butanone	78–93-3	292 g/l (20° C)	Exposure lab	-	24	n.a.^p^	189 ppm	10 ppm	Ratings
		Ethyl benzene	100–41-4	0.17 g/l (25° C)					98 ppm	10 ppm	
[40]	Seeber et al. (2002)^q^	Acetone	67–64-1	Miscible	Exposure lab	–	16		1000 ppm	0 ppm	SPES
		2-butanone	78–93-3	292 g/l (20° C)		–	24		10–380 ppm	n.a	
		Ethanol	64–17-5	Miscible		–	16		100–1900 ppm	n.a	
		Ethyl acetate	141–78-6	85.3 g/l (20° C)		–	16		400 ppm	0 ppm	
		Ethyl benzene	100–41-4	0.17 g/l (25° C)		–	24		10–188 ppm	n.a	
		Iso-propanol	67–63-0	Miscible		–	24		35–350 ppm	n.a	
		1-octanol	111–87-5	0.3 g/l (20° C)		–	24		0.2–12 ppm	n.a	
		Styrene	100–42-5	0.24 g/l (20° C)		–	16		20 ppm	0 ppm	
[41]	Pacharra et al. (2016b)	Ammonia	7664–41-7	531 g/l (20° C)	Exposure lab	26	–	23.2 (SD 1.1) and 24.7 (SD1.4)	10 ppm	Increasing from 0 ppm	Saliva, NALF, LMS, LHS^r^
[42]	Andersson et al. (2015)	n-butanol	71–36-3	77 g/l (20° C)	Exposure lab	30	6	44 (SD 14) and 41 (SD 14)	11.5 mg/m3	Increasing from 0 ppm	Borg scale
[43]	Dantoft et al. (2015)										Airway epithelial lining fluid
[44]	Claeson and Andersson (2017)	Acrolein	107–02-8	267 g/l (20° C)	Exposure lab	26	11	42 (SD 13) and 40 (SD 13)	0.07 mg/m3^s^	0 ppm	Rating of eye irritation, BUT, Skin conductance level
[45]	Österberg et al. (2004)	n-butyl acetat	123–86-4	4.3 g/l (20° C)	Exposure lab	54^t^	46^u^	43.7 (SD 9.6)	6 ppm^v^	–	Rating, performance tests
[46]	Nordin et al. (2005)	Pyridine	110–86-1	Miscible	Dynamic olfactometer	25	13	20–34	19% v/v	13% v/v, 15% v/v	ERP, Borg scale
[47]	Azuma et al. (2016)	γ-undecalactone	104–67-6	0.158 g/l (20° C)	Test strips of blotter paper	16		27–65	–	–	NIRS, rating
		Skatole	83–34-1	Insoluble							
[48]	Alobid et al (2014)	Barcelona Smell Test				118		n.a.^w^			Detection, identification

^a^Water solubility (European Pharmacopoeia, 8. Edition) Descriptive term: Very soluble >1000 g/l, Freely soluble 100–1000 g/l, Soluble 33–100, Sparingly soluble 10–33 g/l, Slightly soluble 1–10 g/l, Very slightly soluble 0.1–1 g/l, Practically insoluble < 0.1 g/l ‘Miscible’: misciple in all proportions with the solvent (here, water)

^b^*VAS* Visual analogue scale

^c^*NALF* Inflammatory markers in NAsal Lavage Fluid (NALF)

^d^*OSB* Oriented strand boards

^e^Total VOC exposure (alpha-pinene, delta3-carene and hexanal)

^f^*LMS* Labeled magnitude scale

^g^*SPES* Swedish Performance Evaluation Scale

^h^*BUT* ocular Break-up time

^i^*NMP* Negative mucosa potential

^j^Group for gender and age comparison; additional subjects were tested

^k^*ERP* Event-related potentials

^l^Two institues did the test with different subjects

^m^100% dilution in odorless mineral oil

^n^For ozone; 35-36 ppb limonene (more details in Fadeyi et al. 2013)

^o^In varying exposure, another 20 subjects were exposed under constant exposure

^p^Students

^q^Several substances were investigated in two experiments with different exposure concentrations (highest concentration is mentioned)

^r^*LHS* Labeled Hedonic Scale

^s^Heptane was used as masking agent and control exposure

^t^Reference group, compared to several other groups (*n* = 158)

^u^Reference group, compared to several other groups (*n* = 142)

^v^Trigeminal irritation is not known to occur below 100 ppm (Österberg et al. 2004)

^w^Older than 18 years

## Abbreviations

AF: Assessment factor
AOP: Adverse outcome pathway
ATP: Adenosine triphosphate
CGRP: Calcitonin gene-related peptide
CI: Chemical intolerance
COPD: Chronic obstructive pulmonary disease
ECHA: European chemicals agency
ERP: Event-related potentials
DMEL/DNEL: Derived minimum effect level/derived no effect level
FPI: Freiburger Persönlickeitsinventar
KE: Key event
LOAEL: Lowest observed adverse effect level
MIE: Molecular initiating event
MCS: Multiple chemical sensitivity
MOA: Mode of action
OA: Occupational asthma
OECD: Organization for economic co-operation and development
OEL: Occupational exposure level
PANAS: Positive affect negative affect scale
PK/PD: Pharmacokinetic/pharmacodynamics modeling
PEL: Permissible exposure limit
QSAR: Quantitative structure–activity relationship
sMCS: Self-reported multiple chemical sensitivity
SPES: Swedish performance evaluation system
TWA: Time weighted average

## Footnotes

1 https://www.merriam-webster.com/thesaurus/vulnerability.

2 The grouping factor is named ‘Vulnerability’ as external factors like chemicals lead to altered perceptions in vulnerable persons. The reason for the altered perception, however, might be an internal factor.

3 Such models comprise of a kinetic component (delivery to target site) and a dynamic component (action at target site and physiological response) in pharmacology or toxicology (Ashauer and Escher 2010).

4 Studies are numbered consecutively. In later tables, studies keep their number for reasons of clarity. In Table 7, all reviewed studies are sorted by number.

5 Water solubility: slightly soluble.

6 Water solubility: slightly soluble.

7 Water solubilities: soluble to freely soluble and miscible.

8 Water solubility: freely soluble.

9 Water solubility: slightly soluble.

10 Water solubility: slightly soluble.

11 Water solubility: very slightly soluble.

12 Water solubility: very slightly soluble.

13 Water solubility: slightly soluble.

14 Water solubility: sparingly soluble.

15 Water solubility: freely soluble.

16 Miscible.

17 Water solubility: very slightly soluble.

18 Water solubilities: soluble to freely soluble and miscible.

19 Water solubility: soluble.

20 Water solubility: freely soluble.

21 Ratings of different symptoms (inter alia nasal and ocular irritation) were conducted on a categorical 6-level- scale.

22 Water solubility: freely soluble.

23 Water solubility: very slightly soluble.

24 Water solubilities: cf., Table 7.

25 Water solubility: freely soluble.

26 Physiology based pharmacodynamic (PBPK) modeling .

27 Pharmacodynamic, pharmacokinetic (PD/PK) modeling.

28 Toxicokinetic, toxicodynamic (TK/TD) modeling.
